# General synthesis of 2,1-benzisoxazoles (anthranils) from nitroarenes and benzylic C–H acids in aprotic media promoted by combination of strong bases and silylating agents

**DOI:** 10.1007/s11030-015-9627-x

**Published:** 2015-08-11

**Authors:** Michał Wiȩcław, Mariusz Bobin, Andrzej Kwast, Robert Bujok, Zbigniew Wróbel, Krzysztof Wojciechowski

**Affiliations:** Institute of Organic Chemistry, Polish Academy of Sciences, Ul. Kasprzaka 44/52, PO Box 58, 01-224 Warszawa, Poland

**Keywords:** Carbanions, Heterocycles, Nucleophilic substitution, Aromatic substitution, Nucleophilic addition, Elimination, Cyanides, Nitroarenes

## Abstract

Carbanions of phenylacetonitriles, benzyl sulfones, and dialkyl benzylphosphonates add nitroarenes at the ortho-position to the nitro group to form $\sigma ^\mathrm{H}$-adducts that, upon treatment with trialkylchlorosilane and additional base (t-BuOK or DBU), transform into 3-aryl-2,1-benzisoxazoles in moderate-to-good yields.

**Electronic supplementary material:**

The online version of this article (doi:10.1007/s11030-015-9627-x) contains supplementary material, which is available to authorized users.

## Introduction

**Scheme 1:**
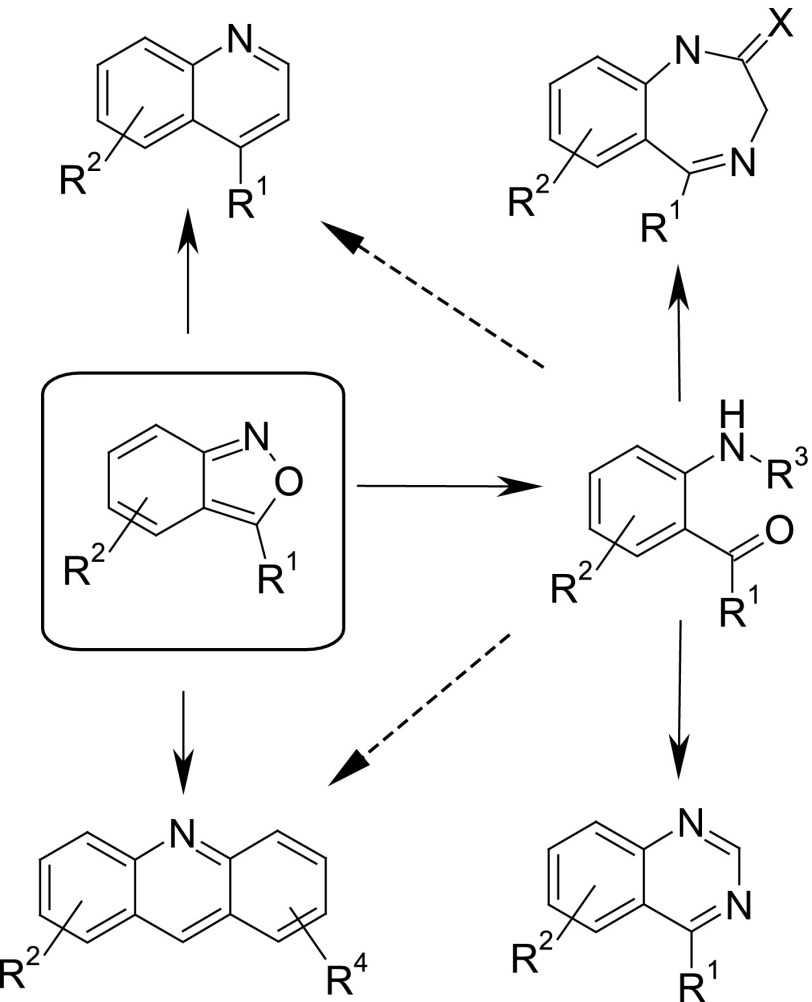
Examples of useful transformations of 2,1-benzisoxazoles

2,1-Benzisoxazoles (anthranils) are important compounds, particularly as starting materials for the synthesis of 2-aminoarylketones [1–4]. A number of heterocyclic systems, such as quinolines, acridines, or quinazolines, can be synthesized either from the latter or, in some cases, directly from 1,2-benzisoxazoles [1–10] (Scheme 1). Of particular interest is the transformation of 3-aryl-2,1-benzisoxazoles into 2-aminobenzophenones, key intermediates in the synthesis of 1,4-benzodiazepines potent psychoactive drugs [11]. Patent literature discloses also a number of anthranils as key intermediates in the synthesis of various drugs, such as mycobacterial agents [12], farnesyl transferase inhibitors [13, 14], protein kinase inhibitors [15], and anticancer agents [16].

**Scheme 2:**
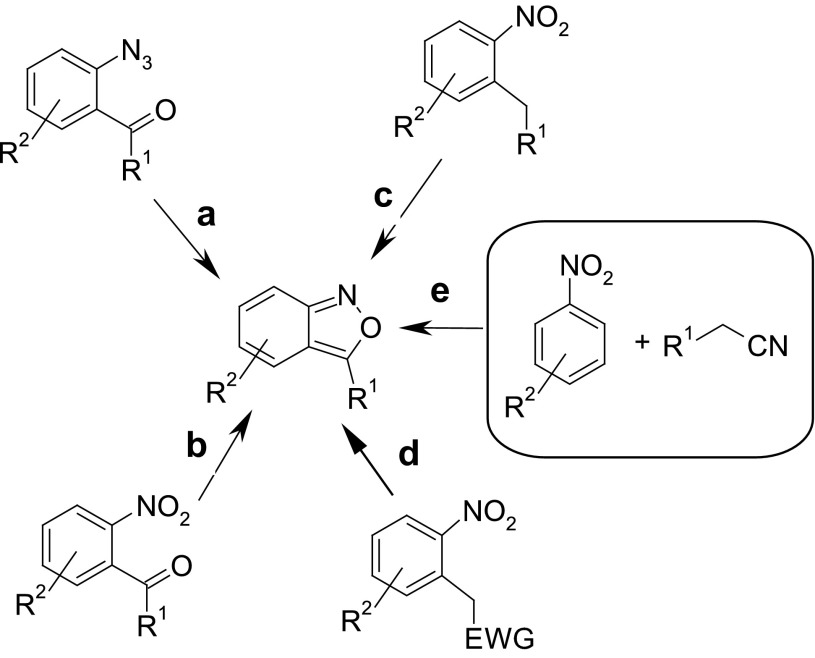
Common methods for the synthesis of 2,1-benzisoxazoles

Numerous methods for the synthesis of 2,1-benzisoxazoles have been developed starting from *ortho*-substituted benzene derivatives containing substituents suitable for cyclization to form a fused isoxazole ring (Scheme 2). The most frequently used are compounds containing such pairs of groups as carbonyl and azido (**a**) [17–20], nitro and carbonyl (**b**) [21–24], and alkyl and nitro (**c**) [25–27]. Dehydration of *ortho*-nitrobenzyl derivatives substituted with electron-withdrawing groups at their methylene unit (**d**) provides anthranils [28–31]. Another approach (**e**), introduced by Davis and Pizzini in 1960 [32], consists of a condensation of nitroarenes and arylacetonitriles, in which the new carbon atom of the isoxazole ring originates from the methylene group of the latter reagent [1, 8, 10, 32, 33].

**Scheme 3:**
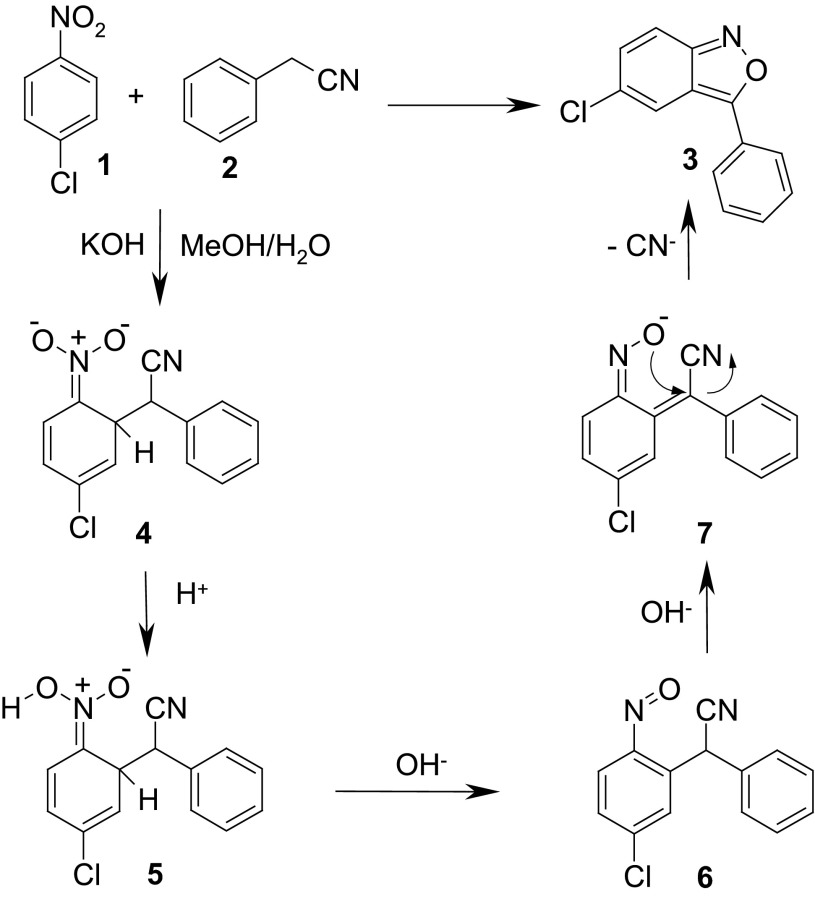
Formation of anthranils from nitroarenes and phenylacetonitriles in protic media

The latter method, although limited to the synthesis of 2-aryl-substituted 2,1-benzisoxazoles, seems to be the most versatile one giving access to the variously substituted 3-aryl-2,1-benzisoxazoles [32]. The whole reaction consists of several reversible steps, and its mechanism is shown in Scheme 3.

According to this mechanism, the reaction proceeds via the formation of $\sigma ^\mathrm{H}$-adduct **4** of the arylacetonitrile anion **2** to the nitroarene **1**. The $\sigma ^\mathrm{H}$-adduct transforms into the nitroso intermediate **6**, which by an intramolecular addition–elimination resulting in the departure of a cyanide anion, forms the isoxazole ring. Protic reaction conditions are crucial for the whole process since the transformation of the $\sigma ^\mathrm{H}$-adduct to the nitroso compound requires a protonation of an oxygen atom of the $\sigma ^\mathrm{H}$-adduct prior to the elimination of the hydroxide ion. The use of protic solvents limits practically the scope of nucleophile precursors to arylacetonitriles although formally some other benzyl derivatives bearing at the $\alpha$-position carbanion-stabilizing groups being also potential leaving groups could be used in this reaction. To such class of compounds belong benzyl sulfides, sulfones and sulfoxides, arylnitromethanes, and dialkyl benzylphosphonates. Another drawback originates from the reversibility of the first reaction step (formation of the $\sigma ^\mathrm{H}$-adduct). The nucleophile must add at position *ortho* to the nitro group to complete the cyclization. It is not a problem if the nitroarene bears at the *para* position a group not prone to substitution. However, when the *para* position contains a hydrogen, a relatively bulky nucleophile forms a thermodynamically more stable $\sigma ^\mathrm{H}$-adduct at this position, which after analogous reaction sequence, leads to methylenequinone-oxime derivatives after elimination of water [32, 34]. On the other hand, there are no literature data for the reaction of *para*-fluoronitrobenzene with arylacetonitriles carbanions proceeding via intermediate $\sigma ^\mathrm{H}$-adducts at *ortho*-position. Probably, if these reactions were attempted, the fluorine atom would be easily substituted leading to 4-nitro-diphenylacetonitrile. For the less electrophilic 4-nitroanisole, only the substitution of the methoxy group was observed [32]. In the literature, there are some examples of nitroarenes with an unoccupied *para* position, which successfully were used in the Davis reaction; however, they should be regarded rather as exceptions [33, 35], but not as a rule [34]. On the other hand, to the best of our knowledge, there are no examples of *ortho*-substituted nitroarenes bearing hydrogen atom at the *para* position, which were used in this reaction.

## Results and discussion

During our studies on the nucleophilic substitution of hydrogen in nitroarenes, we observed that in aprotic solvents, the $\sigma ^\mathrm{H}$-adducts, upon treatment with Lewis acids or silylating agents, transformed into nitroso compounds that further underwent cyclization to afford heterocycles [36–39]. We have found that reactions of nitroarenes with arylacetonitriles or benzyl sulfones performed in DMF in the presence of DBU as a base and $\hbox {MgCl}_{2}$ as a Lewis acid led to the formation of 3-aryl-2,1-benzisoxazole derivatives in moderate-to-good yields [40]. Such “one–pot” approach somewhat broadens the scope of the reaction on the unsubstituted nitrobenzene and some *meta*-substituted nitrobenzenes. The problem of nucleophilic substitution of hydrogen in nitroarenes has been thoroughly studied by Ma̧kosza [41–45], who found that (1) at low temperature, carbanions add very efficiently to nitroarenes furnishing $\sigma ^\mathrm{H}$-adducts almost quantitatively [46–53], and (2) that in relatively low polar solvents, such as THF, the formation of $\sigma ^\mathrm{H}$-adducts occurs predominantly at the *ortho* position to the nitro group. This effect was particularly pronounced in the vicarious nucleophilic substitution (VNS) of hydrogen in nitroarenes by carbanions containing a leaving group attached to a nucleophilic center [54]. We have found that under the right conditions, quenching of the $\sigma ^\mathrm{H}$-adducts at the *ortho*-position to the nitro group with a silylating agent followed by adding a base, in the so-called “step-by-step” procedure, results in the formation of acridines [55] and 3-aminoquinolines [56]. Anthranils were detected as by-products in some experiments during the optimization of the reaction of 4-chloronitrobenzene with phenylacetonitrile leading to acridines [55].

These observations prompted us to investigate the transformations of $\sigma ^\mathrm{H}$ adducts of benzylic carbanions to nitroarenes to find conditions directing the reaction toward the formation of anthranils. Under the standard conditions, a solution mixture of 4-chloronitrobenzene (1 eq) and phenylacetonitrile (1 eq) in dry THF was treated at $-60\,^{\circ } \hbox {C}$ with a solution of t-BuOK (1.1 eq) in THF, stirred for 5 min, then treated with a silylating agent (SA), followed by stirring for 5 min, and finally treated with an additional base (B). Then, the reaction mixture was allowed to warm-up to room temperature and stirred until completion (GC or TLC monitoring). Amounts and types of silylating agent and base are specified in Table 1.

**Table 1:** Optimization of the reaction conditions

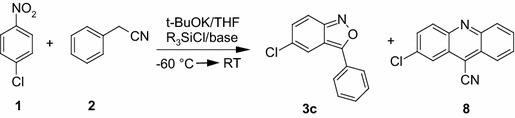							
Entry	Silylating agent		Added base		Time (h)	Product yield (%)$^\mathrm{a}$	
		Amount/eq		Amount/eq		**3**	**8**
1	$\hbox {Me}_{3}\hbox {SiCl}$	5	–	–	24	–	–
2	$\hbox {Me}_{3}\hbox {SiCl}$	2.5	NEt$_{3}$	5	24	–	7
3	$\hbox {Me}_{3}\hbox {SiCl}$	5	NEt$_{3}$	5	24	–	41
4	$\hbox {Me}_{3}\hbox {SiCl}$	5	NEt$_{3}$	10	24	–	50
5	$\hbox {Me}_{3}\hbox {SiCl}$	4	NEt$_{3}$	5	24	Trace	48
6	$\hbox {Me}_{3}\hbox {SiCl}$	1	DBU	5	48	Trace	–
7	$\hbox {Me}_{3}\hbox {SiCl}$	2	DBU	5	1	23	–
8	$\hbox {Me}_{3}\hbox {SiCl}$	3	DBU	5	1	55	–
9	$\hbox {Me}_{3}\hbox {SiCl}$	4	DBU	5	1	78	–
10	$\hbox {Me}_{3}\hbox {SiCl}$	5	DBU	5	1	80	Trace
11	$\hbox {Me}_{3}\hbox {SiCl}$	3	$t\hbox {-BuOK}$	1.1	2	Trace	Trace
12	$\hbox {Me}_{3}\hbox {SiCl}$	3	$t\hbox {-BuOK}$	2	2	8	–
13	$\hbox {Me}_{3}\hbox {SiCl}$	3	$t\hbox {-BuOK}$	4	2	77	–
14	$\hbox {Me}_{3} \hbox {SiCl}$	4	${\textit{t}}$- BuOK	5	2	91	–
15	$t\hbox {-BuMe}_{2}\hbox {SiCl}$	5	$\hbox {NEt}_{3}$	5	120	–	83
16	$t\hbox {-BuMe}_{2}\hbox {SiCl}$	1	DBU	5	24	17	–
17	${{\textit{t}}}{\text {-BuMe}}_{{ 2}}{\text {SiCl}}$	3	DBU	5	1	66	–
18	*t*-BuMe$_{2}$SiCl	2	*t*-BuOK	1.1	48	29	30
19	*t*-BuMe$_{2}$SiCl	3	*t*-BuOK	1.1	48	–	81
20	${\textit{t}}$-BuMe$_{{ 2}}$SiCl	3	${\textit{t}}$-BuOK	2	24	66	–
21	$t\hbox {-BuMe}_{2}\hbox {SiCl}$	5	$t\hbox {-BuOK}$	1.1	24	–	85
22	$t\hbox {-BuMe}_{2}\hbox {SiCl}$	5	$t\hbox {-BuOK}$	2	24	–	50
23	$t\hbox {-BuMe}_{2}\hbox {SiCl}$	5	$t\hbox {-BuOK}$	2.5	24	–	74
24	$t\hbox {-BuMe}_{2}\hbox {SiCl}$	5	$t\hbox {-BuOK}$	5	24	–	Trace

$^\mathrm{a}$ Determined by GC

At the beginning, it was found that the reaction requires an additional base to proceed. This means that the $\sigma ^\mathrm{H}$-adduct, quenched only with a silylating agent, does not react to form anthranil **3** or acridine **8** (entry 1). Then we found that triethylamine was ineffective as a base in reactions leading to anthranil. Regardless of the amount of $\hbox {Et}_{3}\hbox {N}$ and its ratio to the silylating agent (SA), no anthranil **3** was observed, and reactions led to acridine (entries 2–5, 15). DBU gave better results provided its amount exceeded (entries 8, 9, 17) or was equal (entry 10) to the molar amount of the silylating agent, and the best yields were obtained when 3–5 eq. of silylating agent and 5 eq. of DBU were used. A similar tendency was observed when using *t*-BuOK as a base as it gave the best yield of anthranil **3** when 4 eq of $\hbox {Me}_{3}\hbox {SiCl}$ and 5 eq of t-BuOK were employed (entry 14). Again, increasing the ratio of SA to t-BuOK reduced the amount of anthranil **3** This effect was particularly pronounced when *t*-$\hbox {BuMe}_{2}\hbox {SiCl}$ was used as a silylating agent (entries 18–19 and 21–24).

Previously, we used pivaloyl chloride as a reagent for the transformation of $\sigma ^\mathrm{H}$-adducts to quinolines [56] and magnesium chloride for the transformation of $\sigma ^\mathrm{H}$-adducts to anthranils [40]. The attempted use of these reagents instead of the silylating agents in the current studies was unsuccessful. Also no anthranil formation was observed when tetramethylguanidine was used as a base.

**Scheme 4:**
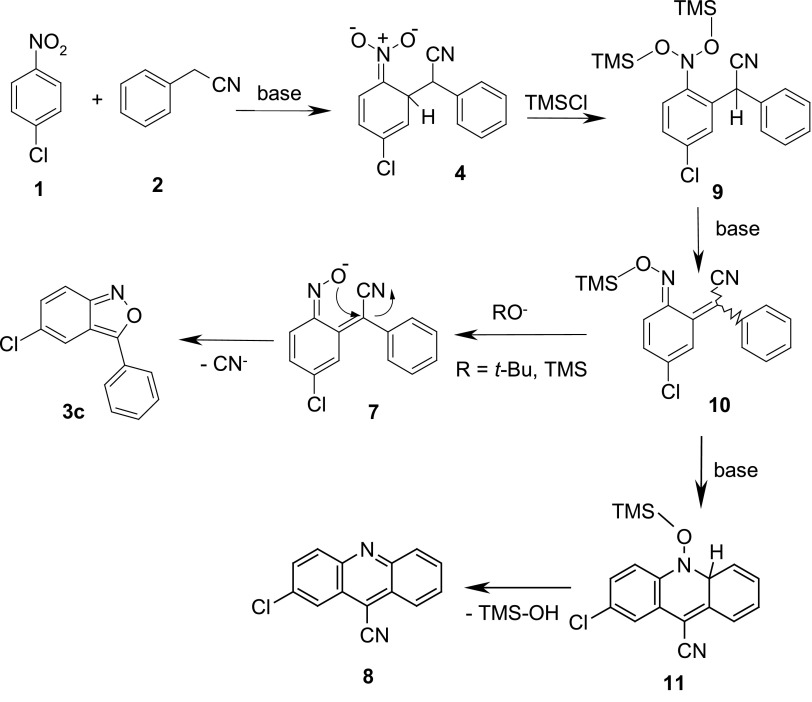
Proposed mechanism for the formation of anthranil and acridine in reactions of $\sigma ^\mathrm{H}$-adduct **4** with trimethylchlorosilane

Analysis of the results presented in Table 1 led us to propose another mechanistic pathway leading to acridines and anthranils (Scheme 4).

**Scheme 5:**
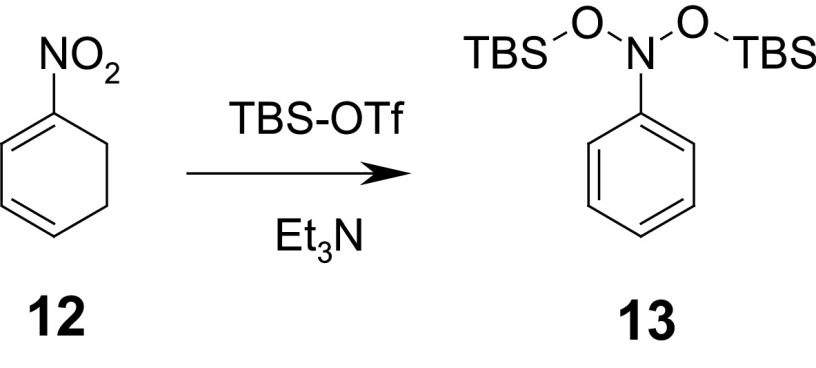
Formation of dihydroxylamine derivative **13**

According to the proposed mechanism, the formation of the intermediate nitroso compound **6** from the $\sigma ^\mathrm{H}$-adduct **4** is abandoned. More plausible seems the bis-silylation of the $\sigma ^\mathrm{H}$-adduct **4** to form the so-called “bis-silylated dihydroxylamine” **9**. Compounds of this type were synthesized by the double deprotonation/silylation of some nitroalkenes [57–60]. The most fitting example is the formation of bis-silylated phenyldihydroxylamine **13** from 1-nitrocyclohexa-1,3-diene (**12**) (Scheme 5) [58].

Formation of anthranils at a higher base/silylating agent ratio could be rationalized as follows. The bis-silylated $\sigma ^\mathrm{H}$-adduct **9**, after 1,4-elimination of silanol anion, gives silylated oxime derivative **10**. Intramolecular electrocyclization of the oxime ether **10** leads, after silanol elimination, to acridine **8**. However, under action of the additional base, particularly *t*-BuOK desilylation of the oxime derivative **10** occurs leading to the nitroso (or oxime) anion **7** which undergoes intramolecular vinylic substitution of the cyano group. This process is facilitated by the presence of an oxygen nucleophile, i.e., in excess of *t*-BuOK, or trialkylsilanol anion generated by DBU or, in much lesser extent, by $\hbox {Et}_{3}\hbox {N}$.

After choosing the most suitable reaction conditions (Table 1, Entry 14), a series of reactions were performed (Table 2). We focused on reactions of such pairs of nitroarene–nucleophile (carbanion), which were not suitable to furnish anthranils under classic conditions proposed by Davis and Pizzini [32, 34, 61]. Unsubstituted nitrobenzene entered the reaction with phenylacetonitrile leading to the formation of 3-phenylbenzisoxazole (**3a**) in moderate yields (entries 1,2). 4-Chloronitrobenzene reacted similarly as under Davis and Pizzini conditions (entries 3–5). To our delight, 4-fluoronitrobenzene successfully participates in the reaction to form expected 5-fluoroanthranils in satisfactory yields (entries 6–8). The observed reaction of 4-fluoronitrobenzene indicates that, at equally activated positions, substitution at the carbon bearing a hydrogen atom is faster than at a carbon bearing any other substituent, including readily replaceable fluorine atom [42, 43, 45].

**Table 2:** Synthesis of 3-arylanthranils

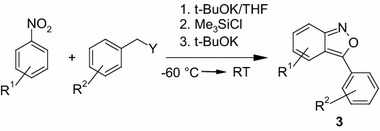						
Entry	$\hbox {R}^{1}$	$\hbox {R}^{2}$	Y	t (h)	Product	Yield (%)$^\mathrm{a}$
1	H	H	CN	2	**3a**	35
2	H	4-MeO	CN	2	**3b**	44
3	4-Cl	H	CN	2	**3c**	90
4	4-Cl	4-Cl	CN	2	**3d**	34
5	4-Cl	4-MeO	CN	2	**3e**	66
6	4-F	H	CN	2	**3f**	49
7	4-F	4-Cl	CN	2	**3g**	49
8	4-F	4-F	CN	2	**3h**	35
9	$4\hbox {-CF}_{3}$	H	CN	2	**3i**	39
10	2-Cl	H	CN	2	**3j**	22
11	4-MeO	4-Cl	SPh	5	**3k**	48
12	4-Cl	H	$\hbox {SO}_{2}$Tol	2	**3c**	46
13	4-Cl	H	$\hbox {PO(OMe)}_{2}$	2	**3c**	22
14	4-MeO	H	$\hbox {PO(OMe)}_{2}^\mathrm{b}$	5	**3l**	35
15	4-$\hbox {Me}_{2}\hbox {N}$	H	$\hbox {PO(OMe)}_{2}^\mathrm{b}$	5	**3m**	15
16	2,4-$(\hbox {MeO})_{2}$	H	$\hbox {PO(OMe)}_{2}^\mathrm{b}$	5	**3n**	52

$$\mathrm{^a}$$ Isolated

$\mathrm{^b}$ Reaction in DMF-THF (4:1) mixture

As we expected, under the above conditions, the reaction was not limited to arylacetonitriles as nucleophile precursors. Interestingly, 4-nitroanisole furnished anthranils **3k** and **3l** when anions of benzyl sulphide (entry 11) or benzylphosphonate (entry 14) were used.

In the case of benzylphosphonate carbanion, the use of a DMF–THF mixture was beneficial, particularly when nitroarenes being weak electrophiles, such as 4-nitroanisole (entry 11), 4-nitro-N,N-dimethylaniline (entry 15), and 2,4-dimethoxynitrobenzene (entry 16) were used. These examples show how robust our new procedure is for the synthesis of anthranils, particularly since these nitroarenes are inactive in reactions with nucleophiles. In the literature, we found only one example of the VNS reaction of 4-nitro-N,N-dimethylaniline with chloromethyl phenyl sulfone (13 % yield) [62] and one example of the Wohl–Aue reaction of 2,4-dimethoxy-1-nitrobenzene leading to the formation of a phenazine derivative in 3 % yield [63].

The reaction of 2-chloronitrobenzene with phenylacetonitrile (entry 10) deserves an additional comment. This reaction leads to the expected anthranil **3j** in moderate yield, and its formation is accompanied by products arising from an oxidation of $\sigma ^\mathrm{H}$-adduct formed at *para* or *ortho* position to the nitro group, most probably 3-chloro-4-(or -2-)-nitrodiphenylacetonitrile.

## Conclusions

We have found that reactions of carbanions with nitroarenes in aprotic conditions using a strong base and silylating agent, 3-arylbenzisoxazoles are formed in good yields. The reaction is general with respect to both nitroarenes and C–H acids activated by groups of nucleofugal character. This reaction does not require a transition metal catalyst and thus can be attractive for use in the pharmaceutical industry.

## Experimental section

All reactions were run under argon atmosphere. Melting points are uncorrected. $^{1}\hbox {H}$ and $^{13}\hbox {C}$ NMR spectra were recorded on a Bruker (500 MHz) (500 MHz for $^{1}\hbox {H}$ and 125 MHz for $^{13}$C spectra), a Varian-NMR-vnmrs600 (600 MHz for 1H spectra) and a Varian Mercury 400 (400 MHz for $^{1}\hbox {H}$ and 100 MHz for $^{13}\hbox {C}$ spectra) instruments. Chemical shifts $\delta$ are expressed in ppm referred to TMS (internal standard), and coupling constants in Hertz (s $=$ singlet, d $=$ doublet, t $=$ triplet, m $=$ multiplet, etc). Mass spectra (EI, 70 eV, and HR-MS) were obtained on a Waters AutoSpec Premier spectrometer. GC analyses were performed on a Hewlett Packard HP6890 GC system with HP5 column and FID (carrier gas—helium). Silica gel Merck 60 (230–400 mesh) was used for flash column chromatography.

### General procedure for optimization of reaction conditions (Table 1)

To a stirred solution of 4-nitrochlorobenzene (157 mg, 1 mmol), phenylacetonitrile (117 mg, 1 mmol) and diphenylsulfone (60 mg, 0.27 mmol, GC internal standard) in THF (5 mL) cooled to $-60\,^{\circ }\hbox {C}$, a solution of t-BuOK (0.13 g, 1.1 mmol) in THF (5 mL) was added. After stirring for 5 min, chlorotrialkylsilane (amount given in Table 1) was added, and the reaction mixture was stirred for another 5 min at this temp. Then, a base (amount given in Table 1) was added. In reactions with additional t-BuOK, it was dissolved in THF (10 mL). The reaction mixture was allowed to reach room temp, and then it was stirred for another 2 h. The final reaction mixture was poured into diluted HCl and extracted with ethyl acetate ($3 \times 10$ mL) and dried with $\hbox {MgSO}_{4}$. The amount of product was determined by GC.

### General procedure for synthesis of anthranils

To a stirred solution of nitroarene (3 mmol) and carbanion precursor (3 mmol) in THF (10 mL) cooled to $-60\,^{\circ }\hbox {C}$, a solution of t-BuOK (0.37 g, 3.3 mmol) in THF (5 mL) was added. After 5 min, chlorotrimethylsilane (1.3 g, 12 mmol) was added, and the reaction mixture was stirred for further 5 min at this temp. Then, t-BuOK (1.68 g, 15  mmol) in THF (20 mL) was added, then the reaction mixture was allowed to cool to room temp, and it was stirred for another 2–5 h. The reaction mixture was then poured into diluted HCl and extracted with ethyl acetate ($3 \times 25$ mL). The combined organic phase was dried with $\hbox {Na}_{2}\hbox {SO}_{4}$. After evaporation of the solvent, the residue was chromatographed (Silica gel, hexane–ethyl acetate 5:1) to afford the desired product. The following compounds were obtained.

#### 3-Phenyl-2,1-benzisoxazole (**3a**)

Yellow solid, yield: 0.21 g (35 %). Mp. 48–50 $^{\circ }\hbox {C}$; (lit. [21] 51–53 $^{\circ }\hbox {C}$). $^{1}\hbox {H}$ NMR (400 MHz, $\hbox {CDCl}_{3})$: $\delta$ $=$ 7.05–7.09 (1 H, m), 7.31–7.35 (1 H, m), 7.48–7.63 (4 H, m), 7.83–7.86 (1 H, m), 8.01–8.04 (2 H, m).

#### 3-(4-Methoxyphenyl)-2,1-benzisoxazole (**3b**)

Yellow crystals, yield: 0.30 g (44 %). Mp. 97–99 $^{\circ }\hbox {C}$; (lit. [64] 99–99.5 $^{\circ }\hbox {C}$). $^{1}\hbox {H}$ NMR (500 MHz, $\hbox {CDCl}_{3})$: $\delta$ $=$ 3.89 (3 H, s), 7.01 (1 H, dd, *J* = 9.0, 6.5 Hz), 7.04–4.09 and 7.95–7.99 (4 H, AA’XX’), 7.30 (1 H, dd, *J* $=$ 9.0, 6.5), 7.57 (1 H, d, *J* $=$ 9.0 Hz), 7.78 (1 H, d, *J* = 9.0 Hz). $^{13}\hbox {C}$ NMR (125 MHz, $\hbox {CDCl}_{3})$: $\delta$ = 55.45, 113.59, 114.74, 115.29, 120.78, 121.25, 123.99, 128.19, 130.56, 157.84, 161.20, 164.6. MS (*m/z*, %): 225 ($\hbox {M}^{+}$, 100), 210 (16), 182 (68), 154 (25), 135 (5), 127 (100). HRMS for $\hbox {C}_{14}\hbox {H}_{11}\hbox {NO}_{2}$ calcd.: 225.0790; found: 225.0798.

#### 5-Chloro-3-phenyl-2,1-benzisoxazole (**3c**)

Yellow solid, yield: 0.62 g (90 %). M.p. 110–112 $^\circ \hbox {C}$ (lit. [32] 115–117 $^{\circ }\hbox {C}$). $^{1}\hbox {H}$ NMR (500 MHz, $\hbox {CDCl}_{3})$: $\delta$ $=$ 7.26 (1 H, dd, *J* $=$ 9.5, 1.8 Hz), 7.50–7.54 (1 H, m), 7.55–7.61 (3 H, m), 7.83–7.84 (1 H, m), 7.96–7.99 (2 H, m).

#### 5-Chloro-3-(4-chlorophenyl)-2,1-benzisoxazole (**3d**)

Yellow crystals, yield: 0.27 g (34 %). M.p. 212–214 $^\circ \hbox {C}$ (lit. [32] 214–215 $^{\circ }\hbox {C}$). $^{1}$H NMR (500 MHz, $\hbox {DMSO-d}_{6})$: $\delta$ $=$ 7.46 (1 H, dd, *J* = 9.6, 1.6 Hz), 7.67–7.70 and 8.15–8.18 (4 H, AA’XX’), 7.78 (1 H, d, *J* $=$ 9.6 Hz), 8.26 (1 H, br s). $^{13}\hbox {C}$ NMR (125 MHz, $\hbox {DMSO-d}_{6})$: $\delta$ $=$ 114.55, 117.71, 119.86, 126.11, 128.80, 130.15, 130.35, 133.30, 136.09, 156.31, 163.29. MS (*m/z*, %): 263 ($\hbox {M}^{+}$, 54), 228 (100), 202 (20), 200 (61), 164 (18), 156 (22), 141 (13), 139 (42). HRMS for $\hbox {C}_{13}\hbox {H}_{7}\hbox {Cl}_{2}\hbox {NO}$ calcd. 262.9905, found 262.9912.

#### 5-Chloro-3-(4-methoxyphenyl)-2,1-benzisoxazole (**3e**)

Yellow solid, yield: 0.51 g (66 %). M.p. 145–147 $^\circ \hbox {C}$ (lit. [32] 143 -145 $^\circ \hbox {C}$). $^{1}\hbox {H}$ NMR (500 MHz, $\hbox {CDCl}_{3})$: $\delta$ $=$ 3.89 (3H, s), 7.05–7.08 and 7.89 – 7.92 (4H, AA’XX’), 7.22 (1 H, dd, *J* $=$ 9.6, 1.7 Hz), 7.54 (1 H, d, *J* $=$ 9.6 Hz), 7.77 (1H, br s). $^{13}\hbox {C}$ NMR (125 MHz, $\hbox {CDCl}_{3})$: $\delta$ $=$ 55.47, 113.68, 114.85, 116.93, 119.15, 120.67, 128.14, 129.55, 132.35, 156.27, 161.44, 164.44. MS (*m/z*, %): 259 ($\hbox {M}^{+}$, 100), 244 (20), 224 (20), 218 (25), 216 (73), 188 (19), 181 (6), 153 (14). HRMS for $\hbox {C}_{14}\hbox {H}_{10}\hbox {ClNO}_{2}$ calcd.: 259.0400; found 259.0406.

#### 5-Fluoro-3-phenyl-2,1-benzisoxazole (**3f**)

Yellow solid, yield: 0.31 g (49 %). M.p. $=$ 93–95 $^\circ \hbox {C}$ lit. [18] 96–97 $^{\circ }\hbox {C}$). $^{1}\hbox {H}$ NMR (500 MHz, $\hbox {CDCl}_{3})$: $\delta$ $=$ 7.18 (1 H, ddd, *J* $=$ 9.6, 8.6, 4.5 Hz), 7.40 (1 H, dd, *J* $=$ 8.6, 2.2 Hz), 7.47–7.51 (1 H, m), 7.53–7.59 (2 H, m), 7.64 (1 H, ddd, *J* $=$ 9.6, 4.5 Hz), 7.94 – 7.96 (2 H, m). $^{13}\hbox {C}$ NMR (125 MHz, $\hbox {CDCl}_{3})$: $\delta$ $=$ 102.24 (d, *J* $=$ 25.5 Hz), 113.38 (d, *J* $=$ 11.6 Hz), 118.16 (d, *J* $=$ 9.3 Hz), 123.77 (d, *J* $=$ 31.3 Hz), 126.31, 128.09, 129.32, 130.29, 155.95, 159.37 (d, *J* $=$ 247 Hz), 164.7 (d, *J* = 11.1 Hz). MS (*m/z*, %): 213 ($\hbox {M}^{+}$, 100), 185 (49), 184 (44), 158 (11), 157 (16), 110 (8), 105 (9). HRMS for $\hbox {C}_{13}\hbox {H}_{8}\hbox {FNO}$ calcd.: 213.0590; found: 213.0587.

#### 5-Fluoro-3-(4-chlorophenyl)-2,1-benzisoxazole (**3g**)

Yellow solid, yield: 0.36 g (49 %). M.p. 205–207 $^{\circ }\hbox {C}$. $^{1}\hbox {H}$ NMR (500 MHz, $\hbox {DMSO-d}_{6})$: $\delta$ $=$ 7.46 (1 H, ddd, *J* $=$ 9.6, 9.6, 2.2 Hz), 7.66–7.69 and 8.11–8.14 (4 H, AA’XX’), 7.83 (1 H, dd, *J* = 9.6, 4.8 Hz), 7.91 (1 H, dd, *J* $=$ 9.6, 2.2 Hz). $^{13}\hbox {C}$ NMR (125 MHz, $\hbox {CDCl}_{3})$: $\delta$ $=$ 103.26 (d, *J* $=$ 26.0 Hz), 113.52 (d, *J* $=$ 11.6 Hz), 118.63 (d, *J* $=$ 9.8 Hz), 124.72 (d, *J*  $=$ 31.8 Hz), 126.30, 128.47, 130.10, 135.76, 156.00, 159.6 (d, *J* $=$ 245 Hz), 163.54 (d, *J* $=$ 11.1 Hz). MS (*m/z*, %): 247 ($\hbox {M}^{+}$, 99), 212 (100), 184 (74). HRMS for $\hbox {C}_{13}\hbox {H}_{7}\hbox {ClFO}$ calcld. 247.0200, found 247.0204.

#### 5-Fluoro-3-(4-fluorophenyl)-2,1-benzisoxazole (**3h**)

Pale yellow solid, yield: 0.24 g (35 %). M.p. 161–162 $^{\circ }\hbox {C}$. $^{1}\hbox {H}$ NMR (500 MHz, $\hbox {CDCl}_{3})$: $\delta$ $=$ 7.18 (1 H, ddd, *J* $=$ 9.6, 8.6, 2.2 Hz), 7.22–7.30 (2H, m), 7.34 (1 H, dd, *J* $=$ 8.6, 2.2 Hz), 7.66 (1 H, *J* $=$ 9.6, 4.6 Hz). $^{13}$C NMR (125 MHz, $\hbox {CDCl}_{3})$: $\delta$ $=$ 101.96 (d, *J* $=$ 25.5 Hz), 113.13 (d, *J* $=$ 11.4 Hz), 116.67 (d, *J* $=$ 22.5 Hz), 118.21 (d, *J* = 9.3 Hz), 123.85 (d, *J* $=$ 31.3 Hz), 124.45 (d, *J* $=$ 3.8 Hz), 128.34 (d, *J* $=$ 8.7 Hz), 155.96, 159.46 (d, *J* $=$ 248 Hz), 163.71 (d, *J* = 10.9 Hz), 163.72 (d, *J* $=$ 253 Hz). MS (*m/z*, %): 231 $(\hbox {M}^{+} _{, }$100), 203 (44), 202 (44), 182 (8), 175 (10). HRMS for $\hbox {C}_{13}\hbox {H}_{7}\hbox {F}_{2}\hbox {NO}$ calcd. 231.0496; found 231.0498.

#### 3-Phenyl-5-trifluoromethyl-2,1-benzisoxazole (**3i**)

Yellow crystals, yield: 0.31 g (39 %). M.p. 116–121 $$^{\circ }\hbox {C}$$. $$^{1}\hbox {H}$$ NMR (500 MHz, $$\hbox {CDCl}_{3})$$: $$\delta $$ $$=$$ 7.46 (1 H, dd, *J* $$=$$ 9.5, 1.4 Hz), 7.55–7.63 (3 H, m), 7.73–7.75 (1 H, m), 8.01–8.03 (2 H, m), 8.18–8.19 (1 H, m). $$^{13}\hbox {C}$$ NMR (125 MHz, CDCl$$_{3})$$: $$\delta $$ $$=$$ 112.90, 117.10, 120.13 (q, *J* $$=$$ 5.3 Hz), 123.78 (q, *J* $$=$$ 272 Hz), 125.69 (*J* $$=$$ 2.6 Hz), 126.78 (*J* $$=$$ 32.5 Hz), 126.96, 131.25, 157.49, 167.51. MS (*m/z*, %): 263 $$(\hbox {M}^{+}$$, 100), 244 (12), 235 (14), 216 (10), 194 (6), 185 (9), 166 (21). 51 (22), 77 (52), 105 (14), 166 (21), 216 (10), 244 (12), 263 (100), 264 (26). HRMS for $$\hbox {C}_{14}\hbox {H}_{8}\hbox {F}_{3}\hbox {NO}$$ calcd. 263.0558, found 263.0554.

#### 7-Chloro-3-phenyl-2,1-benzisoxazole (**3j**)

Yellow crystals, yield: 0.15 g (22 %). Mp. 105–$107\,^{\circ }{\hbox {C}}$. $^{1}\hbox {H}$ NMR (500 MHz, $\hbox {CDCl}_{3})$: $\delta$ $=$ 7.00 (1 H, dd, J $=$ 8.8, 7.0 Hz), 7.35 (1 H, d, *J* $=$ 7.0 Hz), 7.50–7.59 (3 H, m), 7.76 (1 H, d, *J* $=$ 8.8 Hz), 8.00–8.02 (2H, m). $^{13}\hbox {C}$ NMR (125 MHz, $\hbox {CDCl}_{3}) \quad \delta$ $=$ 115.56, 119.47, 121.49, 124.81, 126.75, 127.94, 129.37, 129.79, 130.78, 156.22, 166.28. MS (*m/z*, %): 229 $(\hbox {M}^{+}$, 100), 201 (17), 194 (65), 166 (72), 164 (27), 140 (23), 139 (25), 105 (29). HRMS for $\hbox {C}_{13}\hbox {H}_{8}\hbox {ClNO}$ calcd. 229.0294; found 229.0293.

#### 5-Methoxy-3-(4-chlorophenyl)-2,1-benzisoxazole (**3k**)

Pale yellow crystals, yield: 0.37 g (48 %). M.p. 140–$141 \,^{\circ }{\hbox {C}}$. $^{1}\hbox {H}$ NMR (500 MHz, $\hbox {CDCl}_{3})$: $\delta$ $=$ 3.00 (3 H, s), 6.79 (1 H, d, *J* = 2.2 Hz), 7.06 (1 H, dd, *J*  $=$ 9.6, 2.2 Hz), 7.50–7.53 and 7.85–7.88 (4 H, AA’XX’), 7.53 (1 H, d, *J* $=$ 9.6 Hz). $^{13}\hbox {C}$ NMR (125 MHz, $\hbox {CDCl}_{3}) \quad \delta$ $=$ 55.48, 93.73, 114.48, 117.19, 127.20, 127.23, 127.80, 129.50, 135.53, 156.02, 157.01, 160.91. MS (*m/z*, %): 259 $(\hbox {M}^{+}$, 30), 224 (100), 216 (23), 196 (8), 188 (11), 181 (13). HRMS for $\hbox {C}_{14}\hbox {H}_{10}\hbox {ClNO}_{2}$ calcd. 250.0400; found. 259.0400.

#### 5-Methoxy-3-phenyl-2,1-benzisoxazole (**3l**)

Yellow crystals, yield: 0.24 g (35 %). M.p. 79–80 $^{\circ }\hbox {C}$. H$^{1}$HNMR (500 MHz, $\hbox {CDCl}_{3})$: $\delta$ $=$ 3.88 (1 H, s), 6.87 (1 H, d, *J* $=$ 2.2 Hz), 7.05 (1 H, dd, *J* $=$ 9.6, 2.2 Hz), 7.44–7.47 (1 H, m), 7.52–7.56 (3 H, m), 7.93–7.96 (2 H, m). $^{13}\hbox {C NMR}$ (125 MHz, $\hbox {CDCl}_{3})$: $\delta$ $=$ 55.44, 94.11, 114.33, 117.08, 126.12, 127.67, 128.83, 129.19, 129.56, 156.01, 156.75, 162.19. MS (*m/z*, %): 225 $(\hbox {M}^{+}$, 84), 210 (39), 182 (100), 154 (43), 128 (13), 127 (14). HRMS for $\hbox {C}_{14}\hbox {H}_{11}\hbox {NO}_{2}$ calcd. 225.0790; found 225.0797.

#### N,N-dimetyl-3-phenyl-2,1-benzisoxazol-5-amine (**3m**)

Yellow crystals, yield: 0.11 g (15 %). M.p. 113–115 $^{\circ }\hbox {C}$. $^{1}\hbox {H}$ NMR (500 MHz, $\hbox {CDCl}_{3})$: $\delta$ $=$ 3.02 (6 H, s), 7.25 (1 H, dd, *J* $=$ 9.4, 2.4 Hz), 7.40–7.43 (1 H, m), 7.52–7.55 (3 H, m), 7.95–7.97 (2 H, m). $^{13}\hbox {C}$ NMR (125 MHz, $\hbox {CDCl}_{3})$: $\delta$ $=$ 41.35, 94.74, 115.48, 116.29, 125.71, 125.86, 128.97, 129.08, 129.33, 147.44, 155.35 [one signal missing]. MS (*m/z*, %): 238 $(\hbox {M}^{+}$, 100), 237 (49), 223 (24), 209 (12), 195 (40), 167 (20). HRMS for $\hbox {C}_{15}\hbox {H}_{14}\hbox {N}_{2}\hbox {O}$ calcd. 238.1106; found 238.1105.

#### 5,7-Dimethoxy-3-phenyl-2,1-benzisoxazole (**3n**)

Yellow crystals, yield: 0.40 g (52 %). M.p. 151 $^{\circ }\hbox {C}$. $^{1}\hbox {H}$ NMR (500 MHz, $\hbox {CDCl}_{3})$: $\delta$ =3.87 (3 H, s), 3.98 (3 H, s), 6.25 (1 H, d, *J* $=$ 1.6 Hz), 6.46 (1 H, d, *J* $=$ 1.6 Hz), 7.42–7.44 (1 H, m), 7.51–7.54 (2H, m), 7.92–7.94 (2 H, m). $^{13}\hbox {C}$ NMR (125 MHz, $\hbox {CDCl}_{3})$: $\delta$ = 55.58, 55.87, 86.43, 102.51, 115.13, 126.05, 128.79, 129.11, 129.46, 149.09, 151.70, 158.10, 162.16. MS (*m/z*, %): 255 $(\hbox {M}^{+}$, 86), 254 (100), 240 (20), 226 (71), 225 (30), 224 (37), 212 (22), 183 (18), 182 (31), 169 (17). HRMS for $\hbox {C}_{15}\hbox {H}_{13}\hbox {NO}_{3}$ calcd.: 255.0895, found: 255.0883.

## Electronic supplementary material


**Supplementary Material** 1H and 13C spectra of compounds ** 3a**-3**n**. (doc 6.23mb)
