# Supplementary material for: General synthesis of 2,1-benzisoxazoles (anthranils) from nitroarenes and benzylic C–H acids in aprotic media promoted by combination of strong bases and silylating agents
Source: Mol Divers. 2015 Aug 11;19(4):807–16. doi: 10.1007/s11030-015-9627-x (PMC4591207; doi:10.1007/s11030-015-9627-x)

Supporting information

General synthesis of 2,1-benzisoxazoles (anthranils) from nitroarenes and benzylic C-H acids in aprotic media promoted by combination of strong bases and silylating agents

Michał Więcław,^[a]^ Mariusz Bobin,^[a]^ Andrzej Kwast,^[a]^ Robert Bujok,^[a]^ Zbigniew Wróbel,*^[a]^ and Krzysztof Wojciechowski*^[a]^

Institute of Organic Chemistry – Polish Academy of Sciences Ul. Kasprzaka 44/52, POBox 58, 01-224 Warszawa, Poland

E-mail: [zwrobel@icho.edu.pl](mailto:zwrobel@icho.edu.pl); kwojciechowski@icho.edu.pl

**1H and 13C NMR spectra of the obtained compounds**


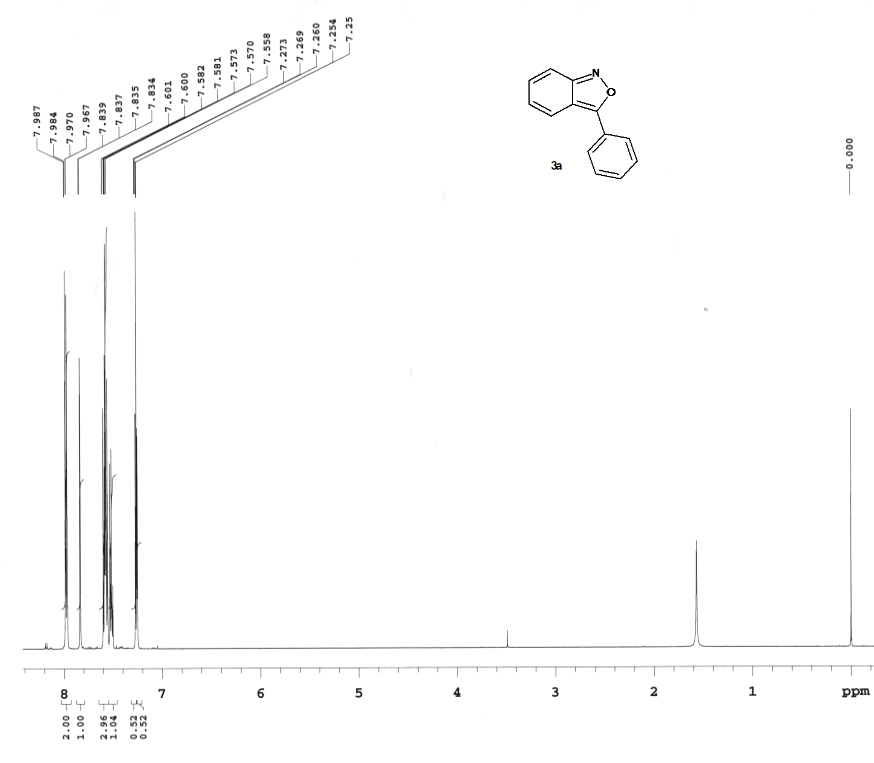

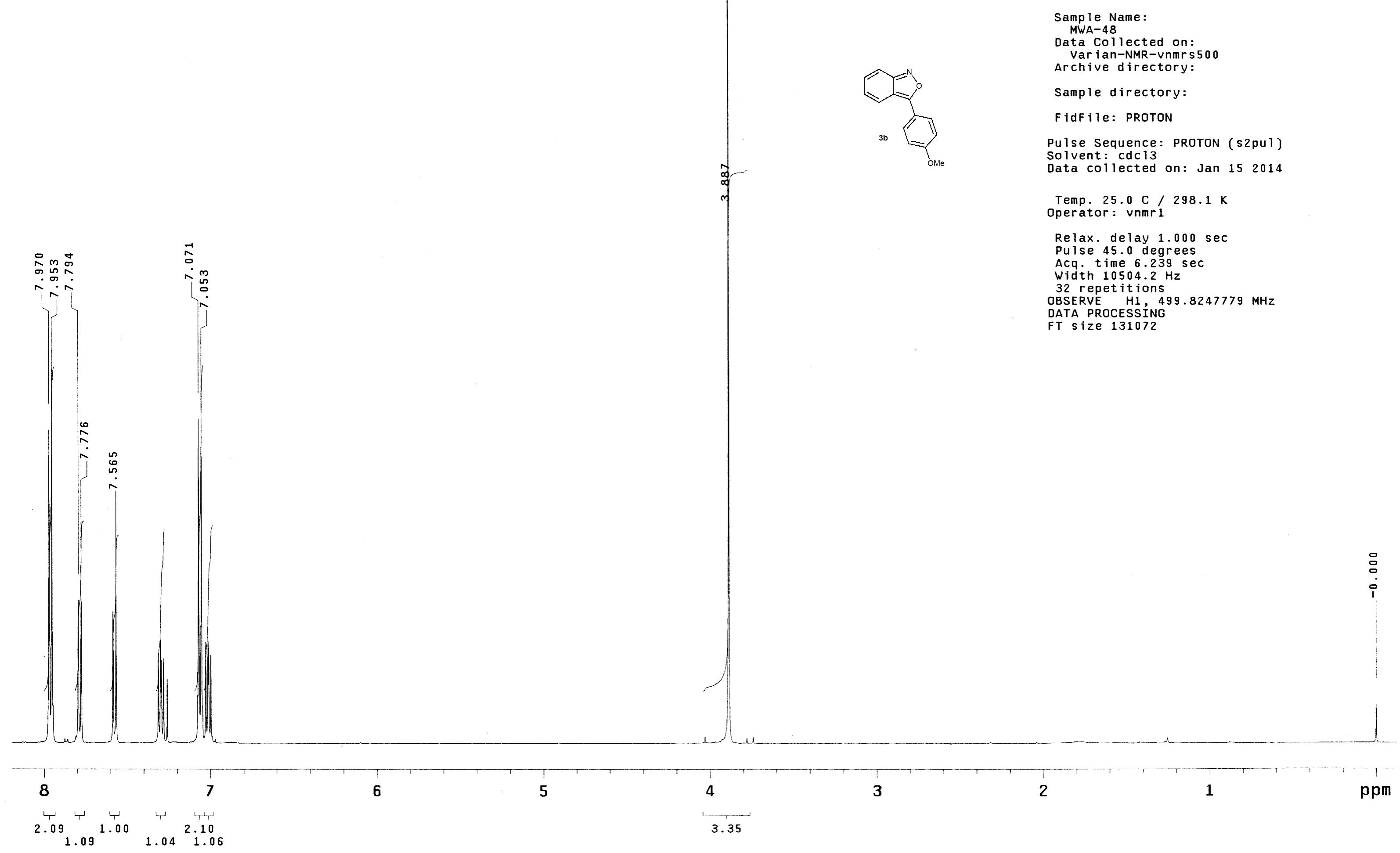


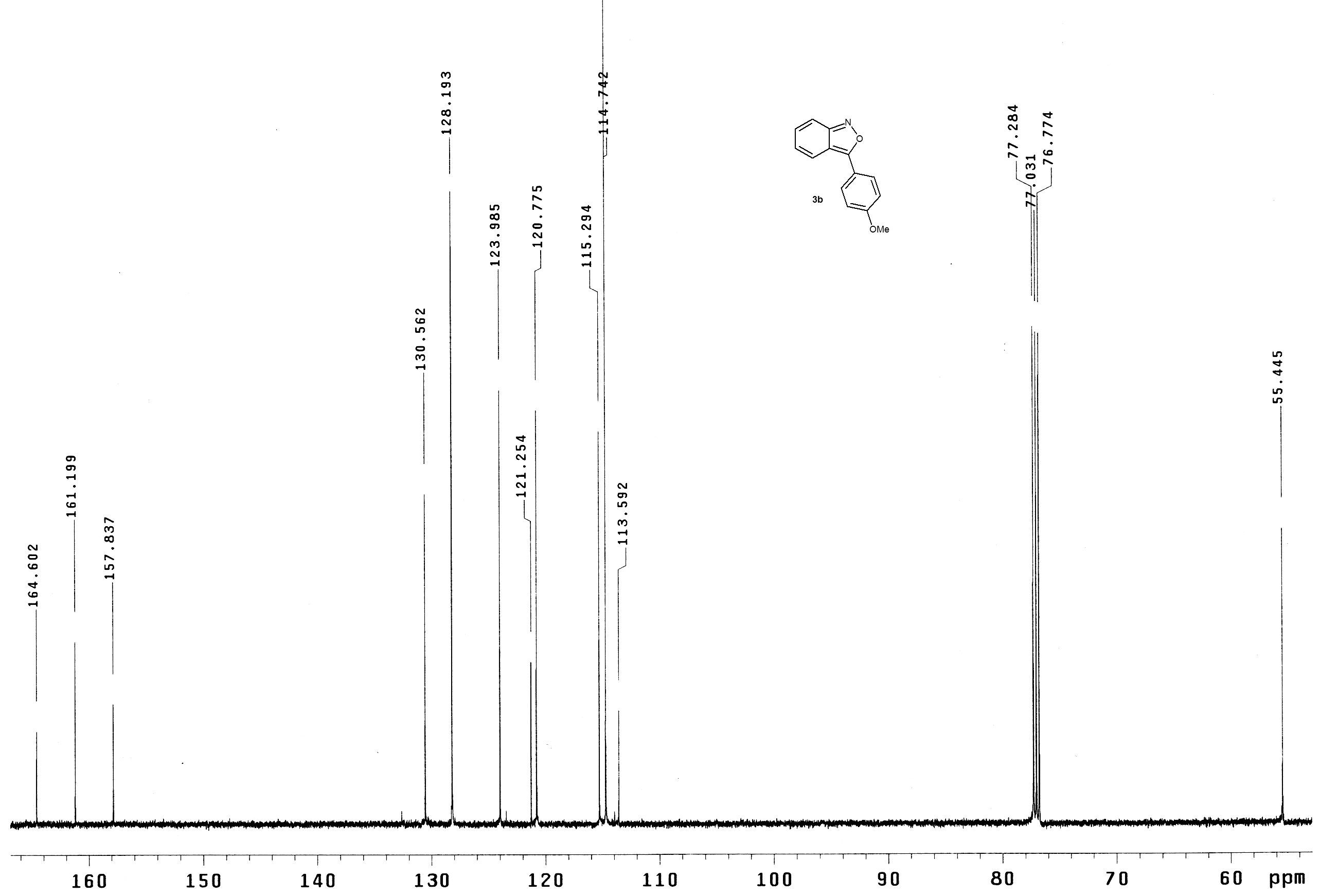

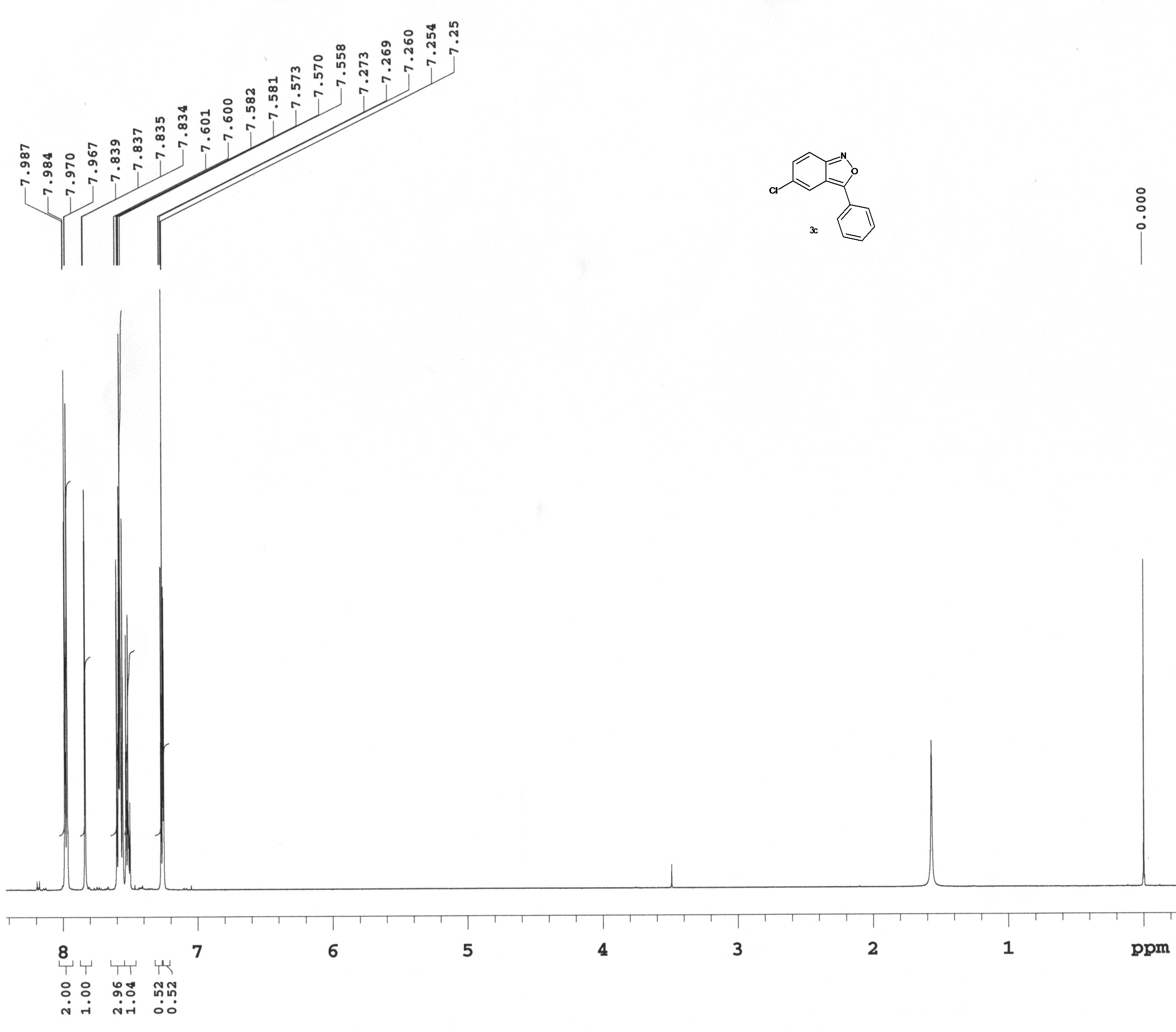

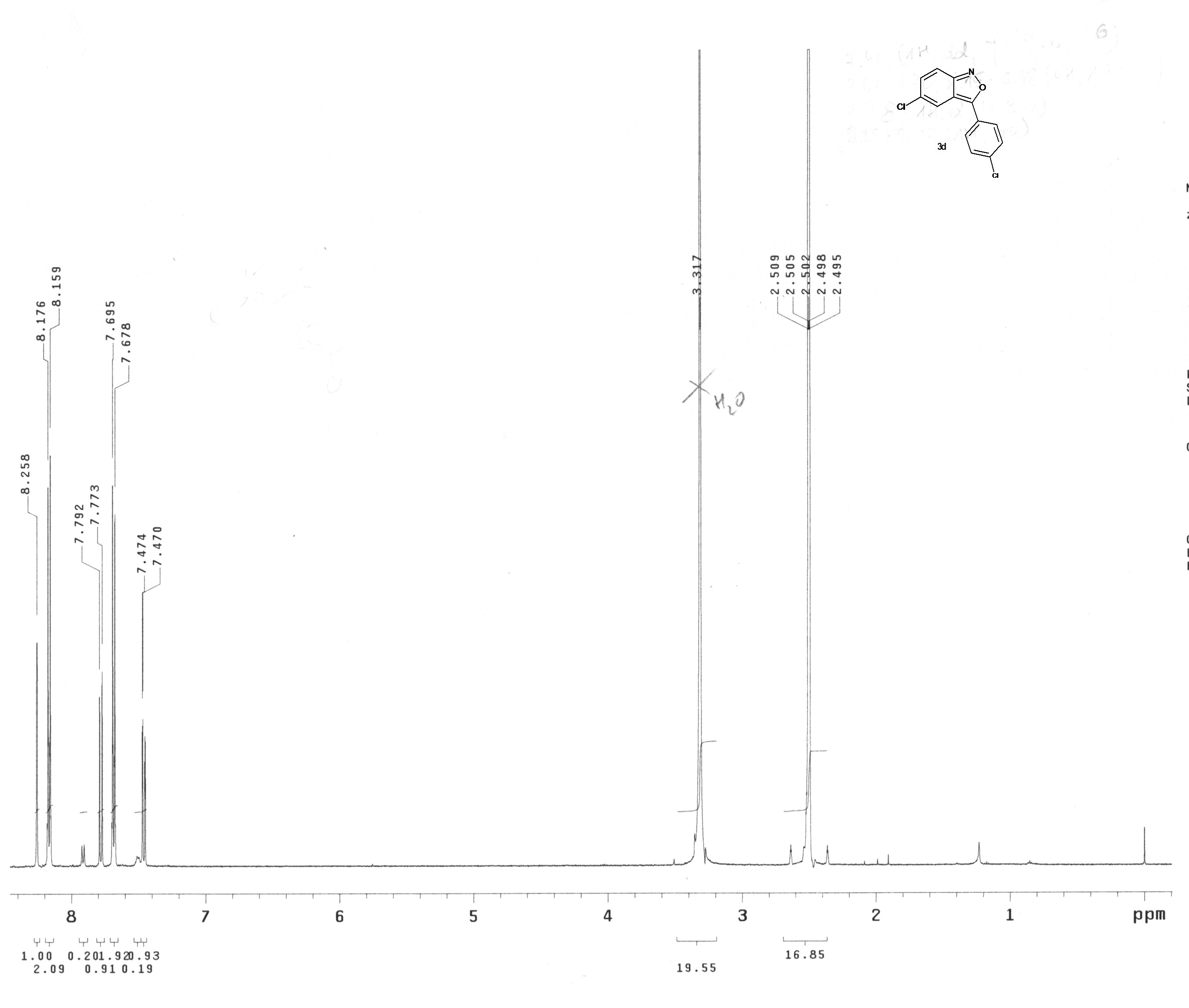

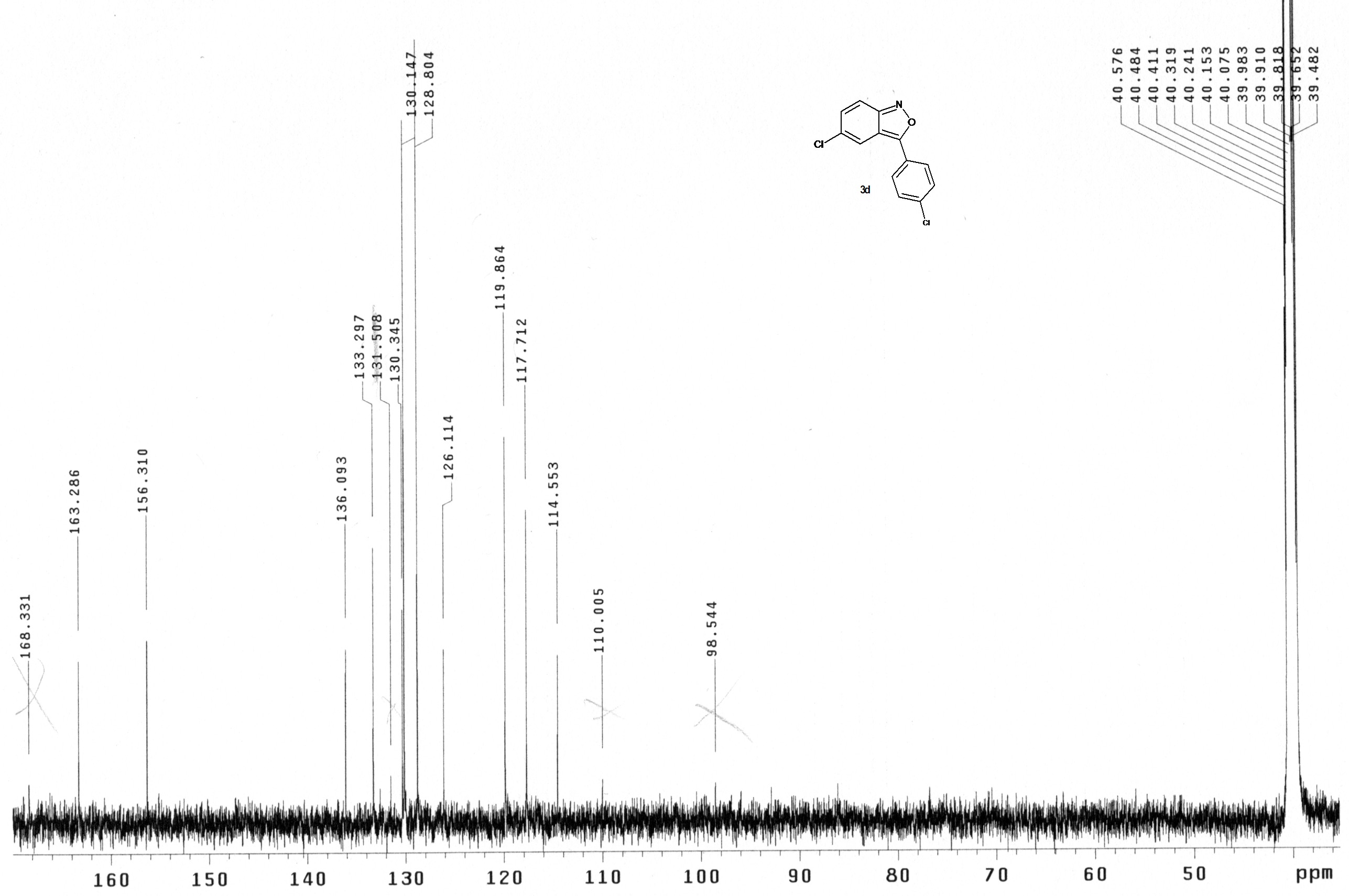

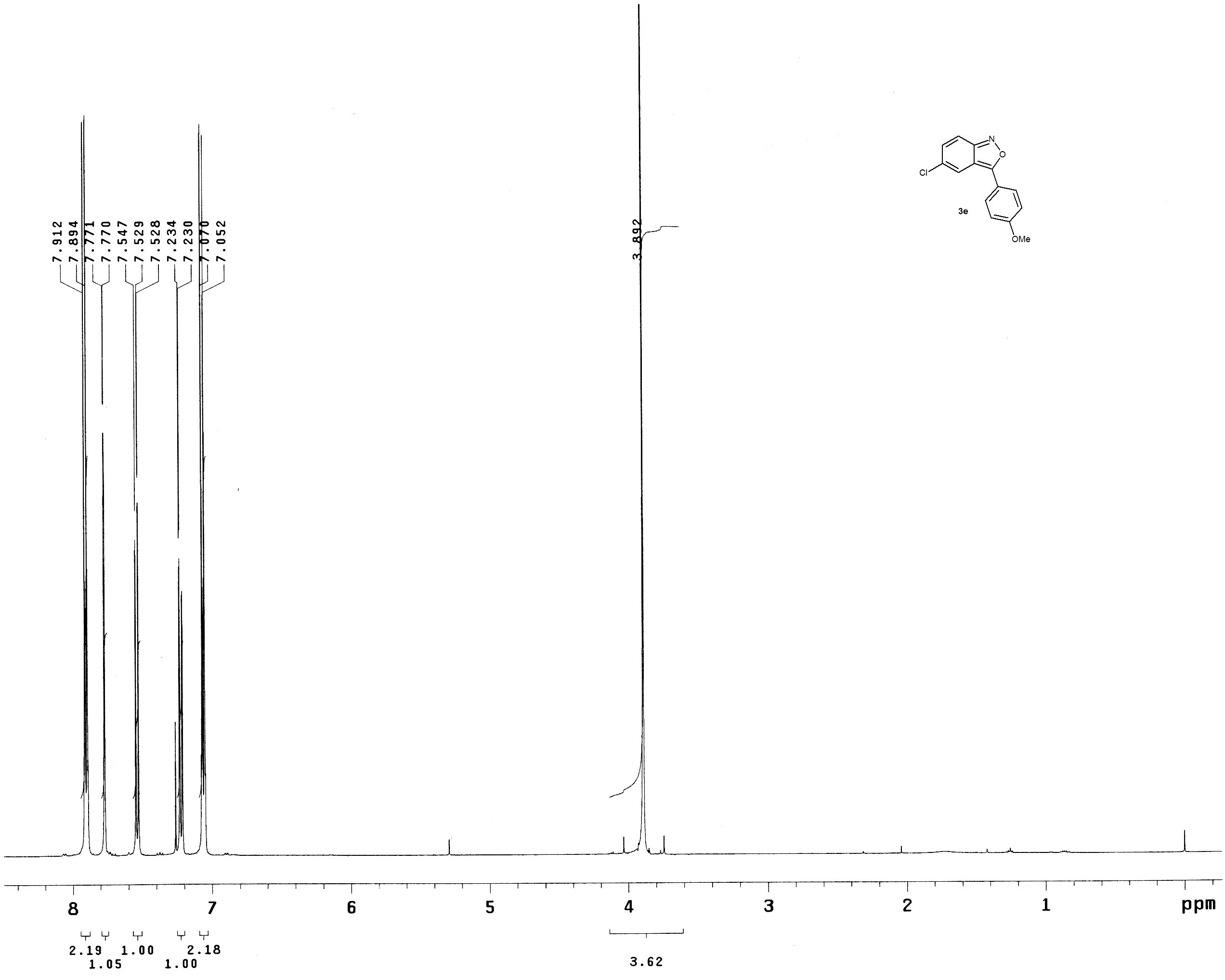

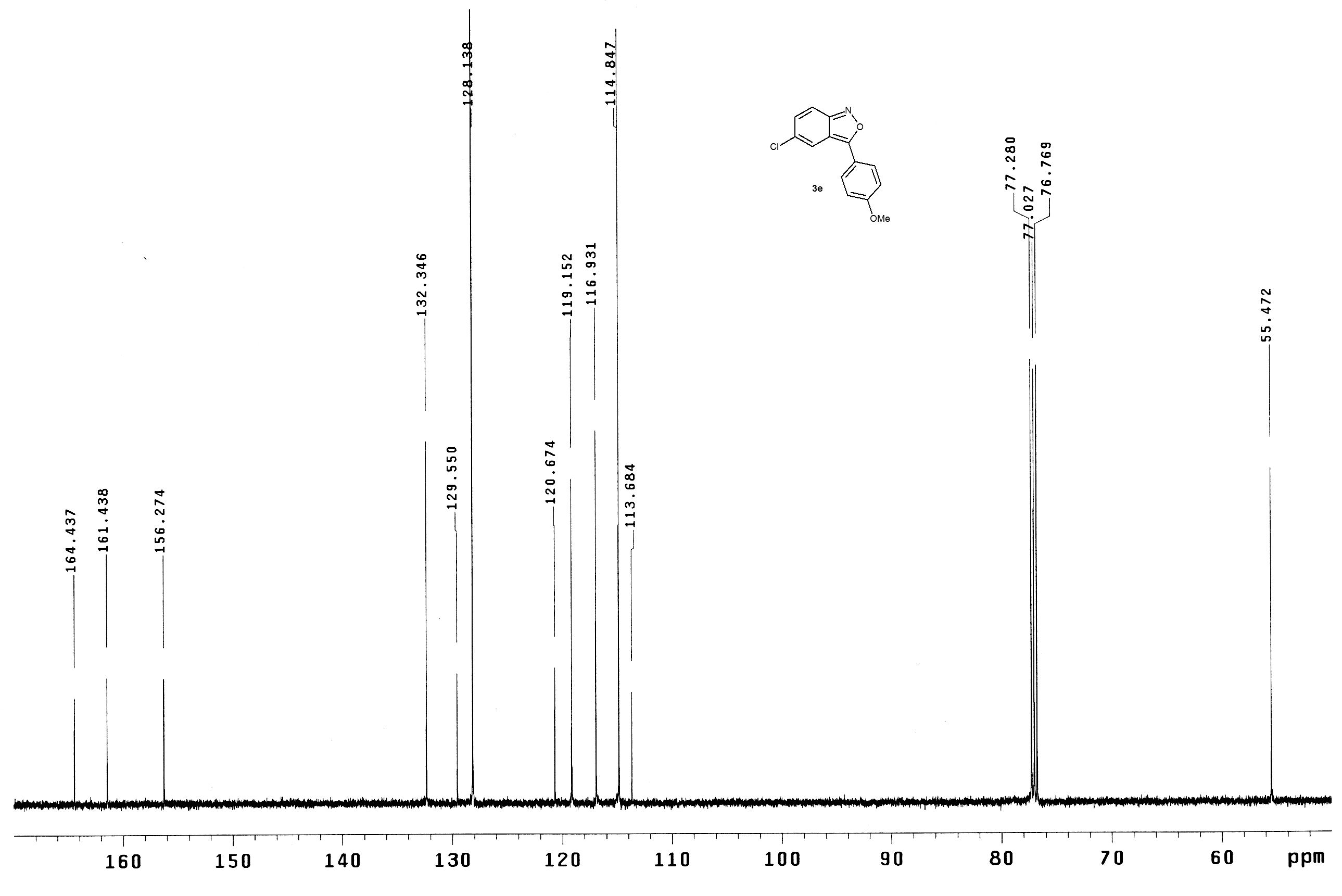

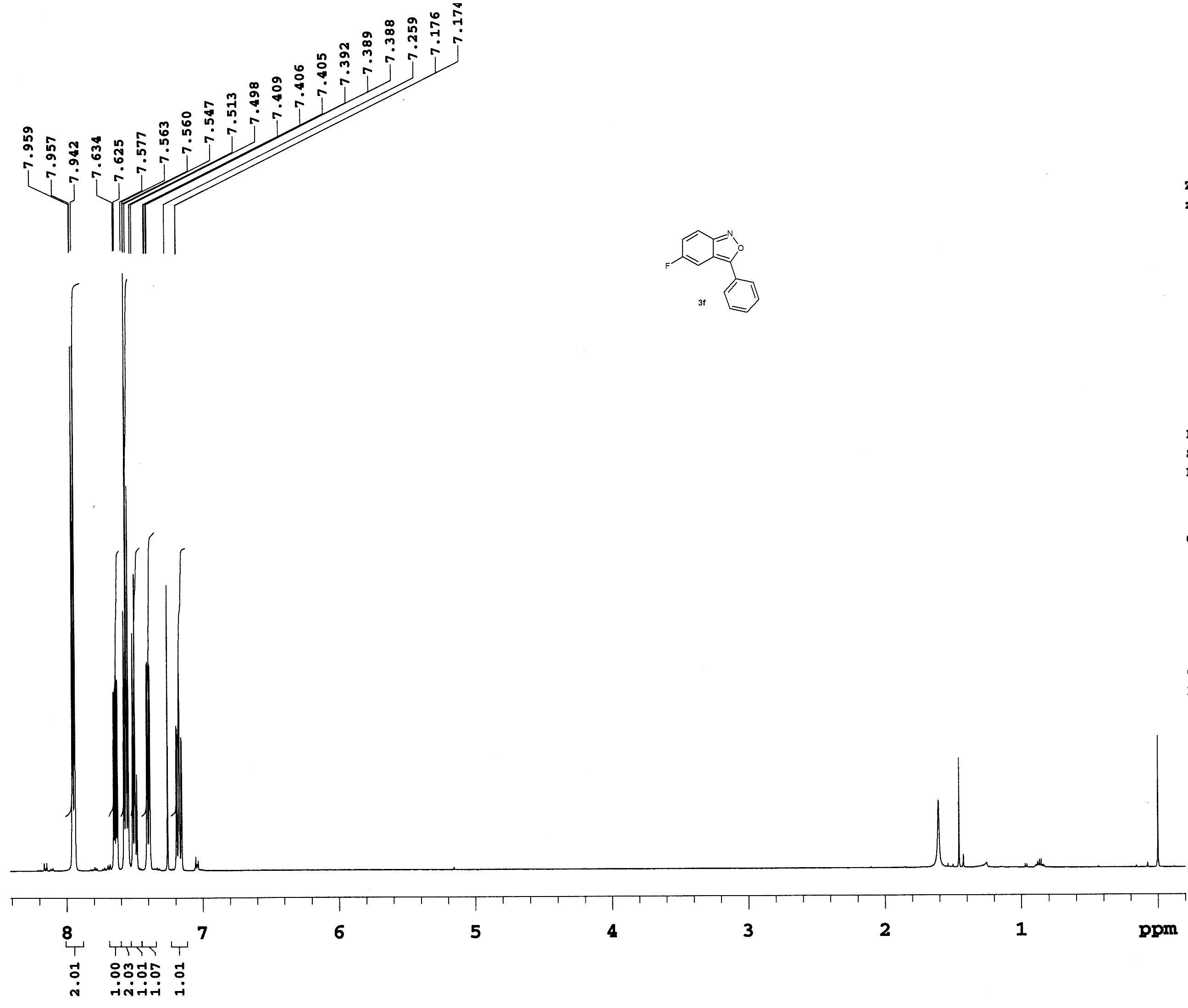

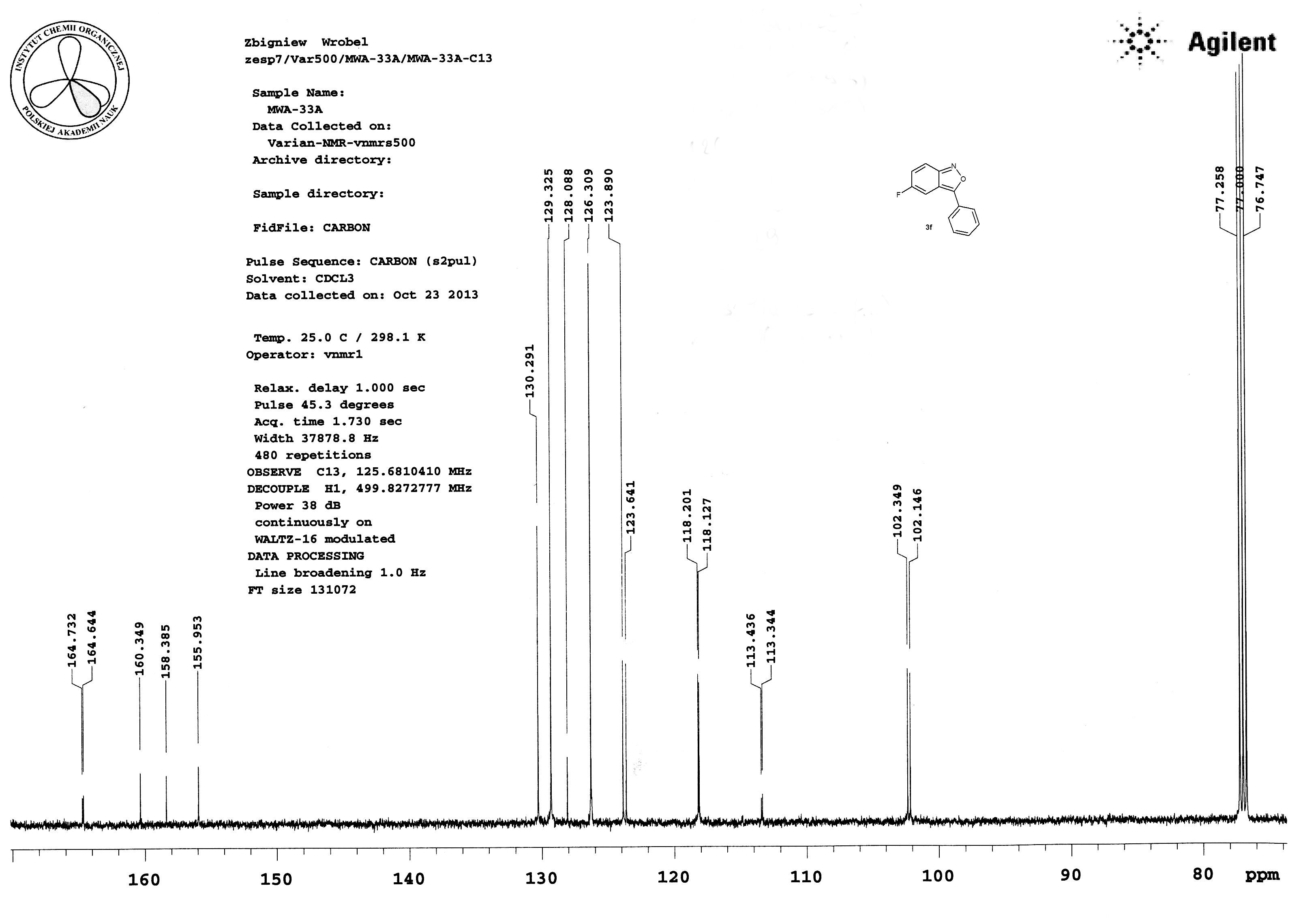


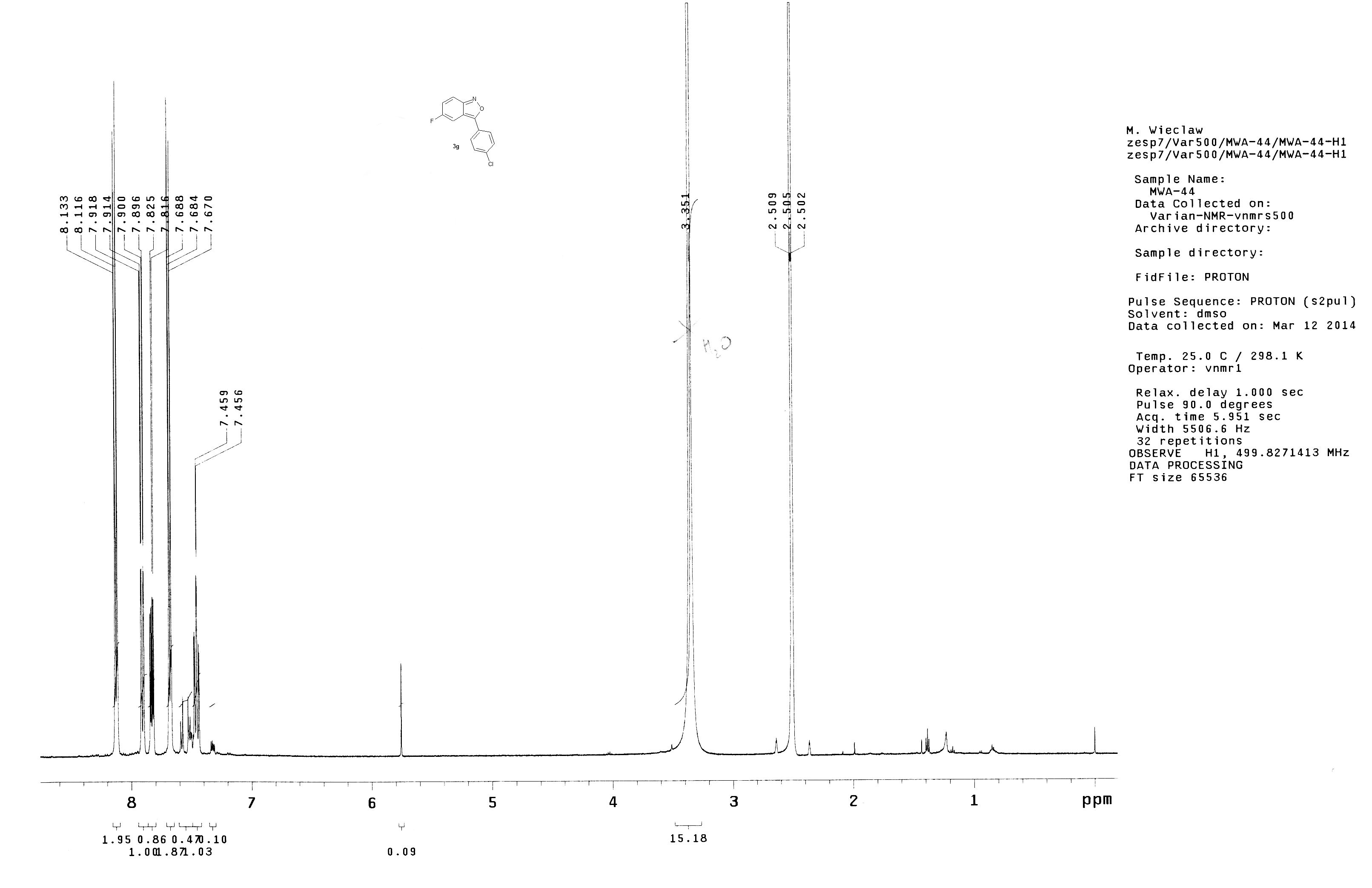


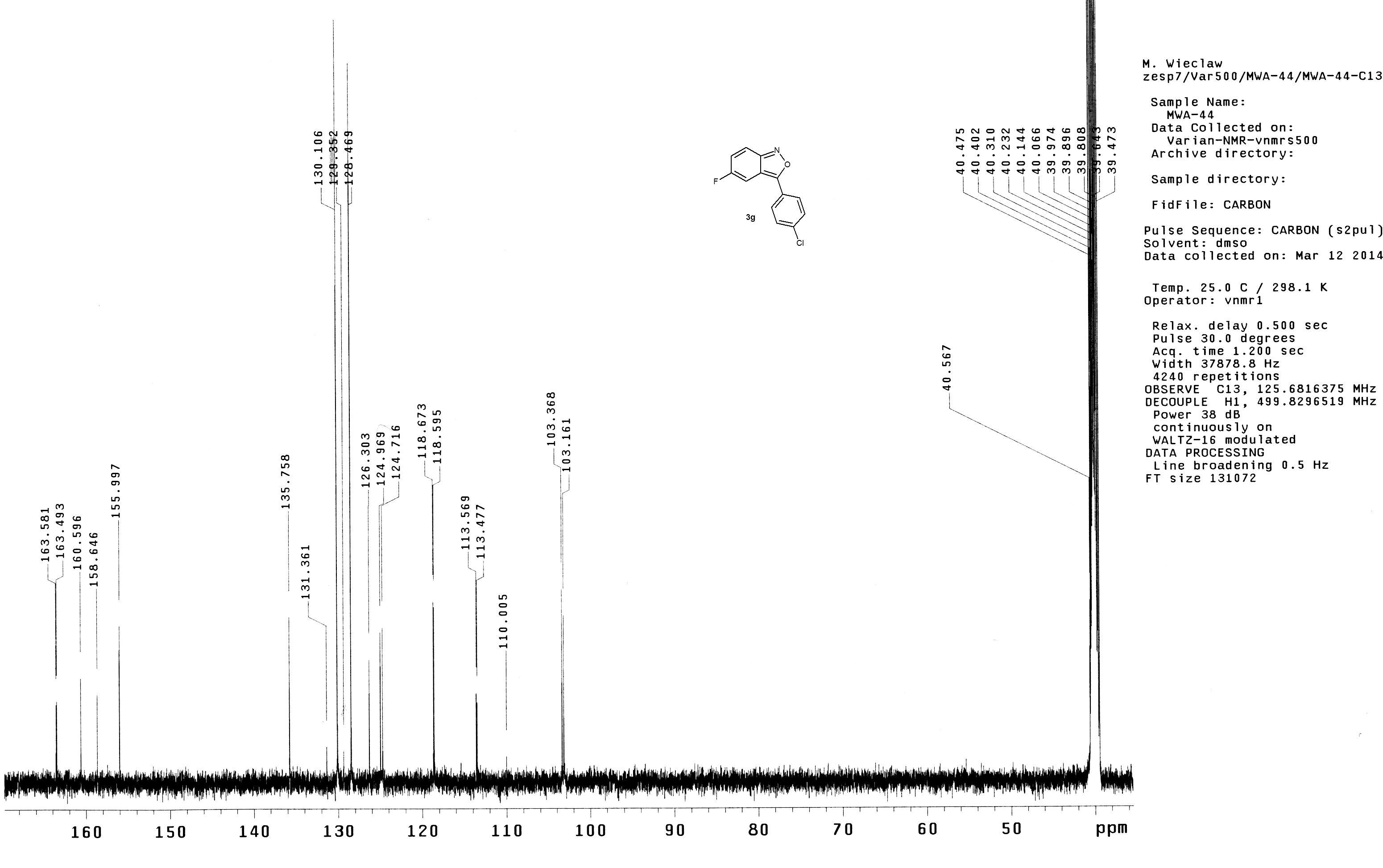


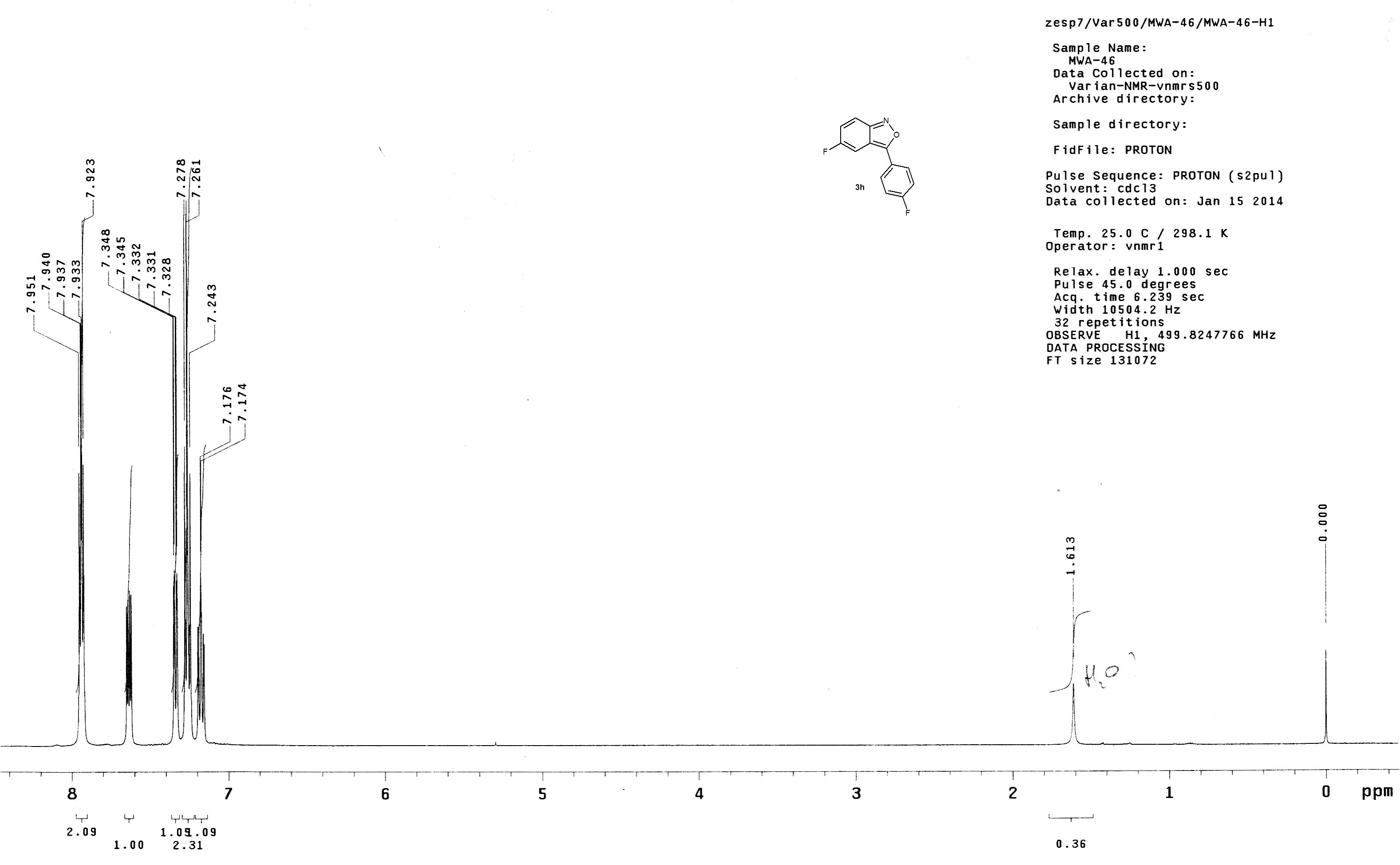


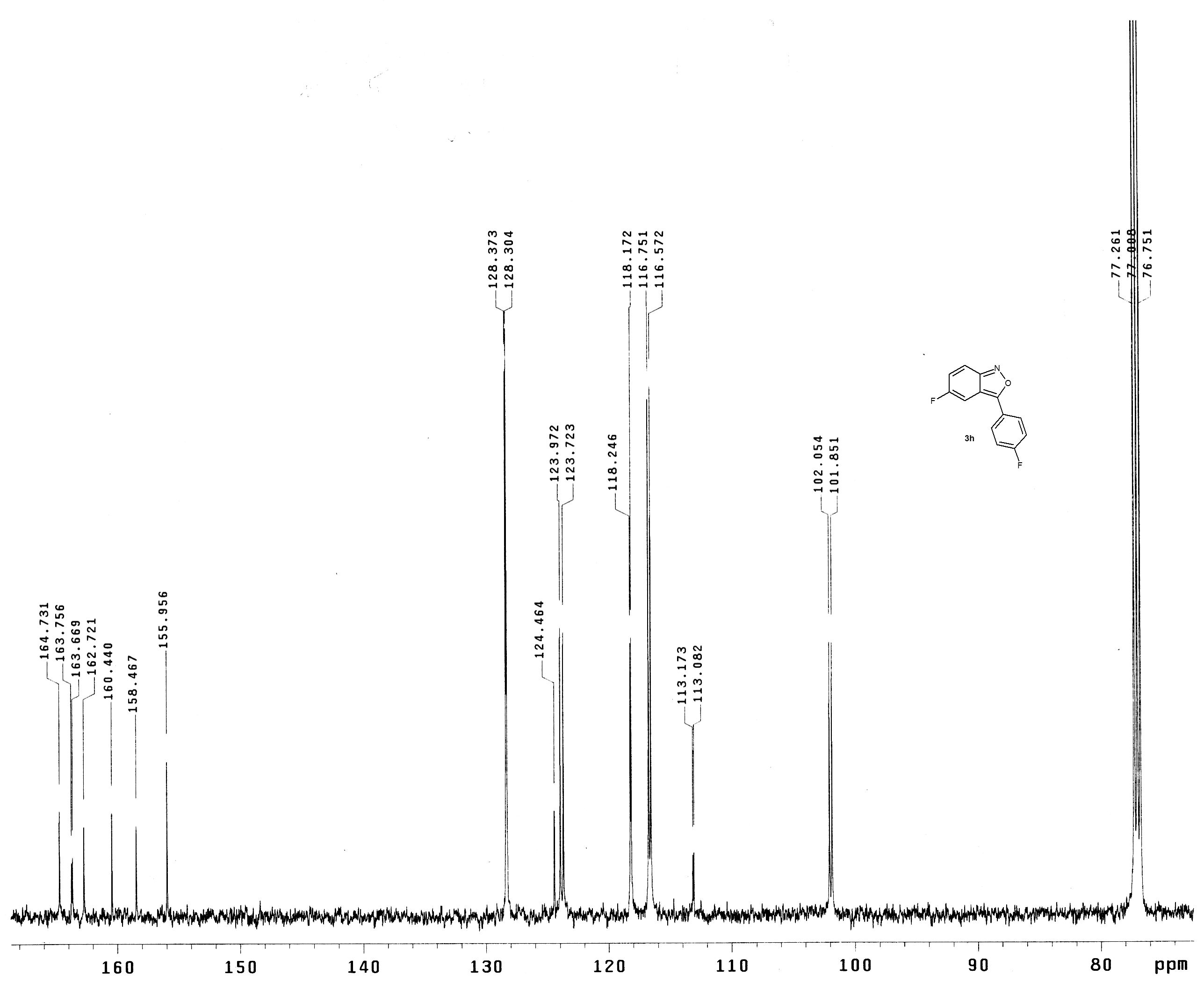


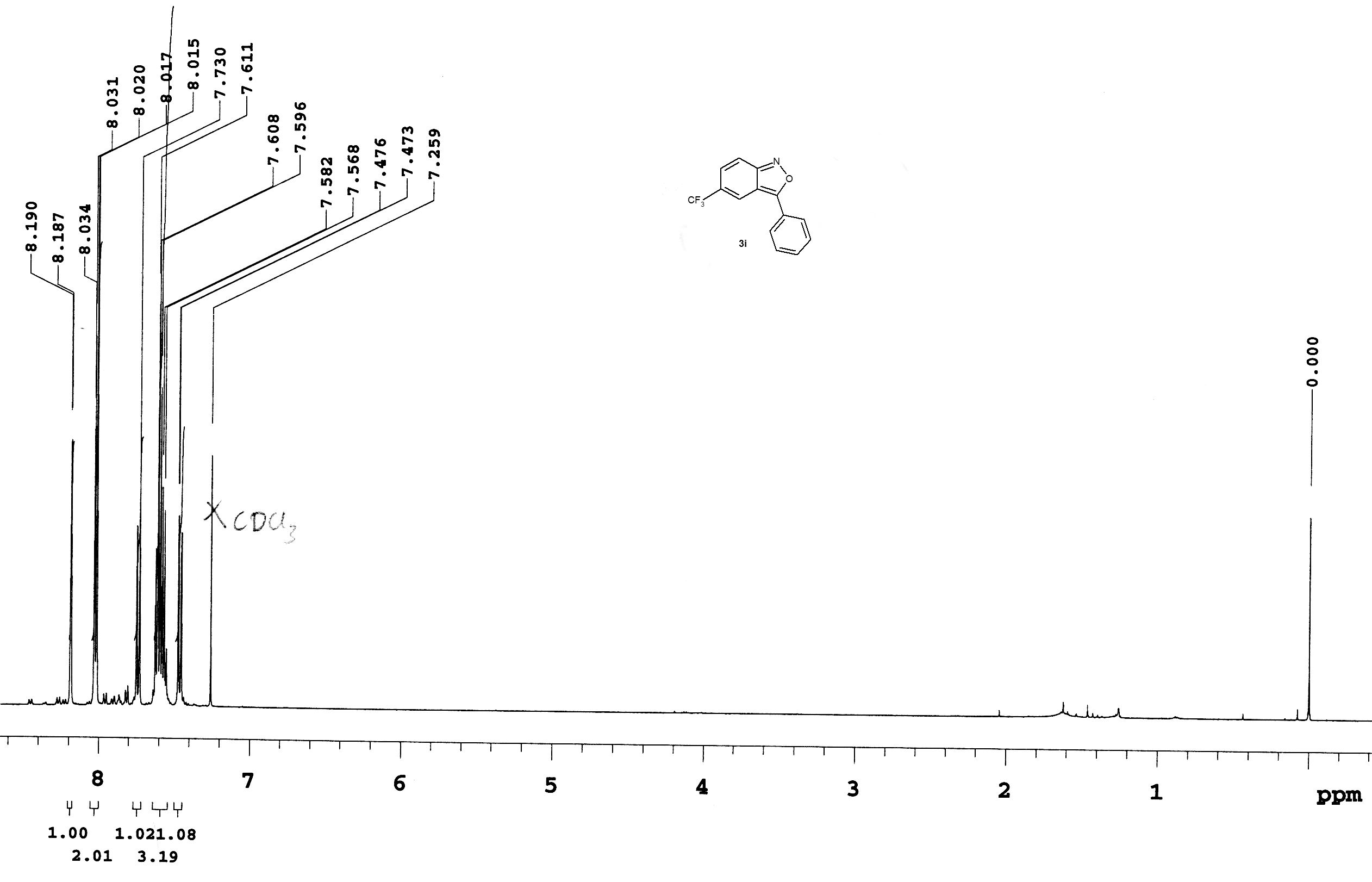

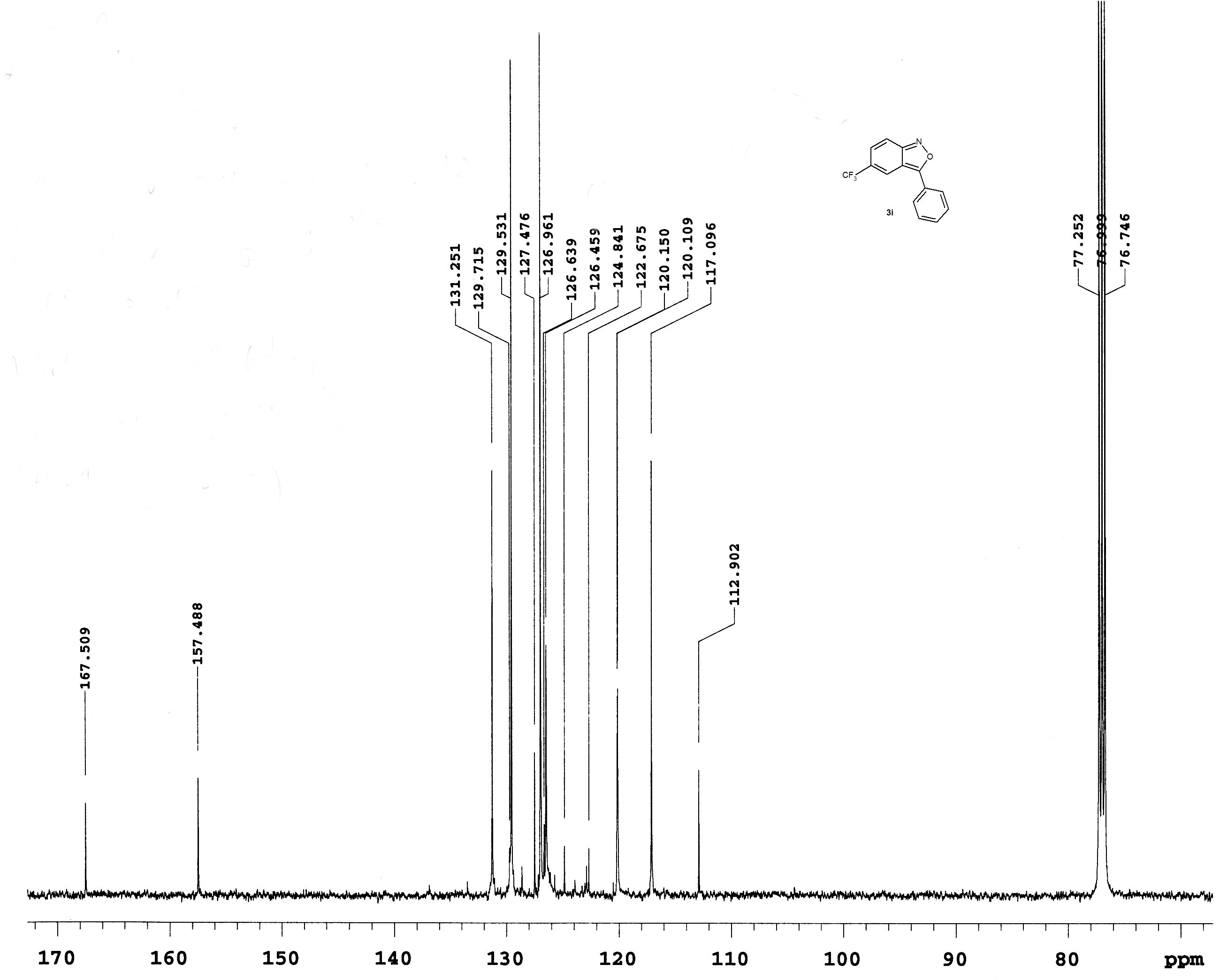

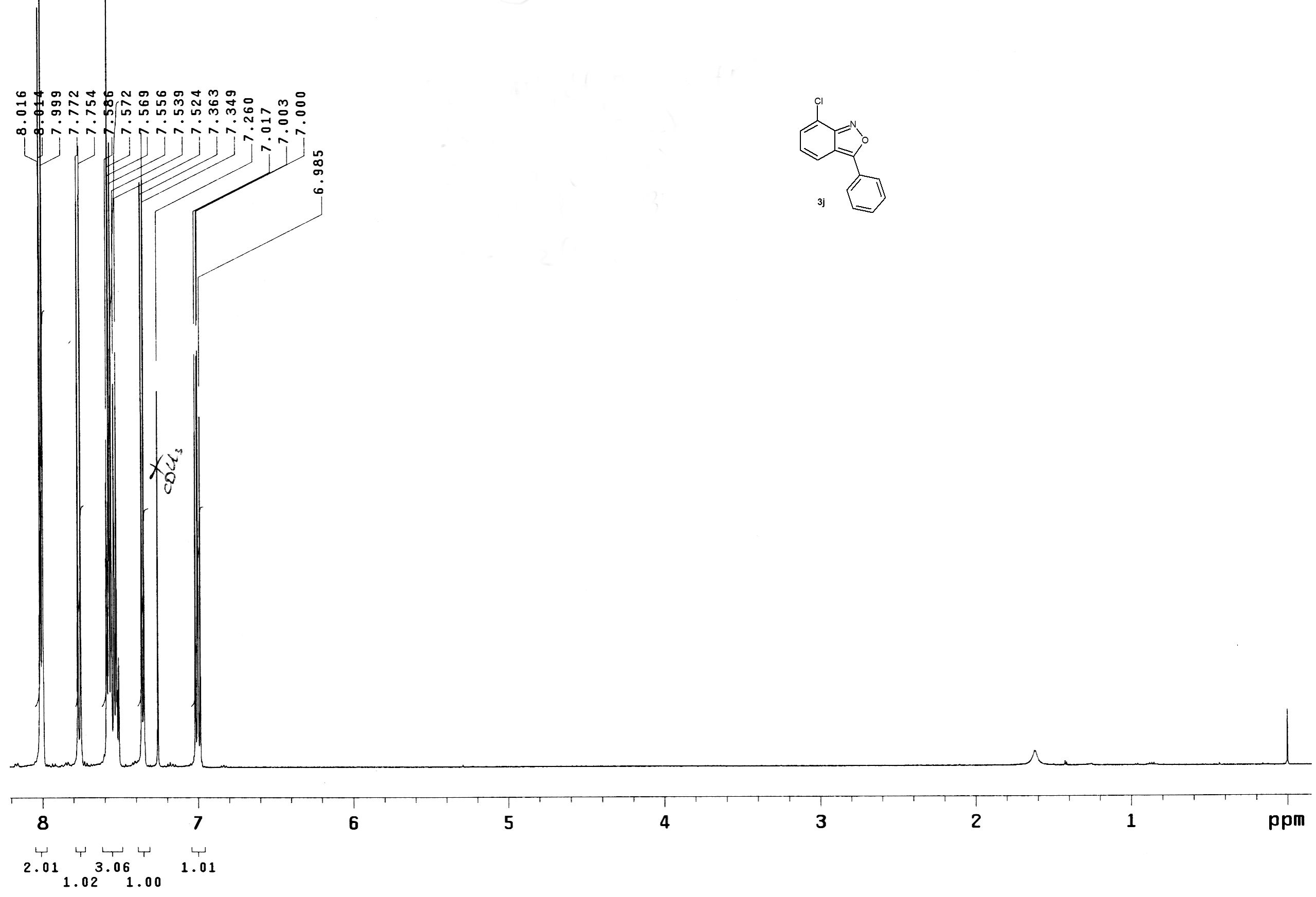

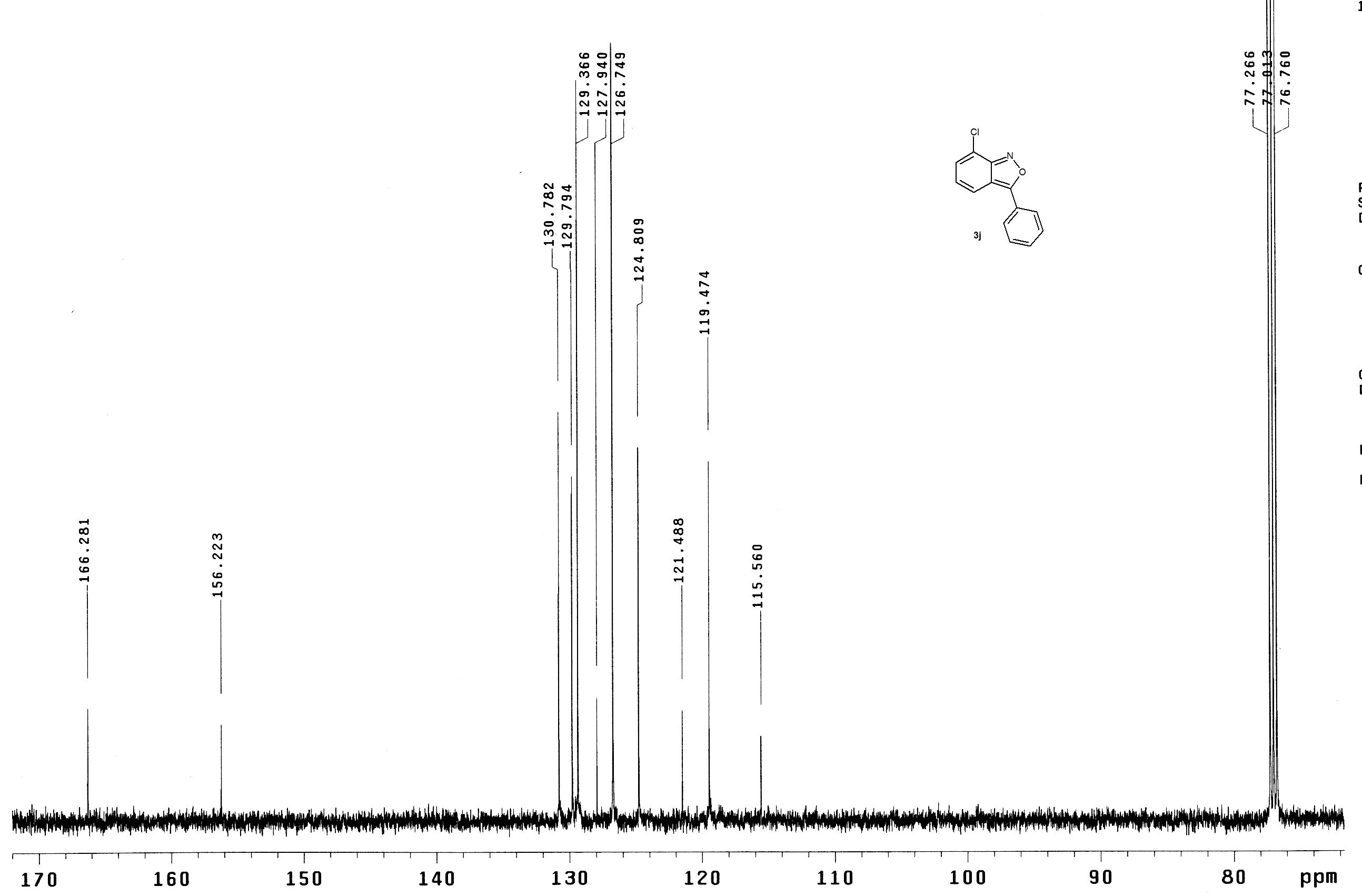

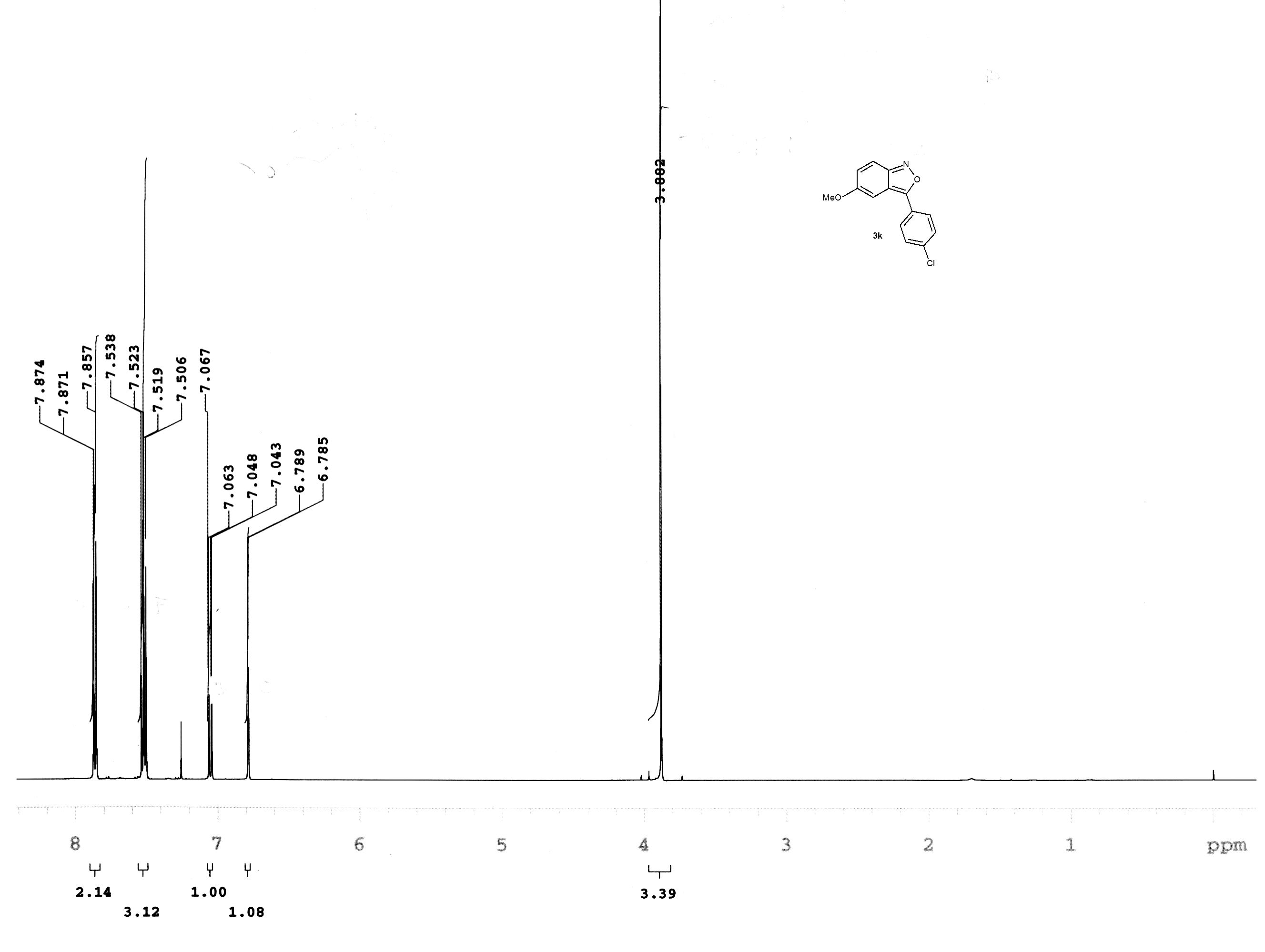

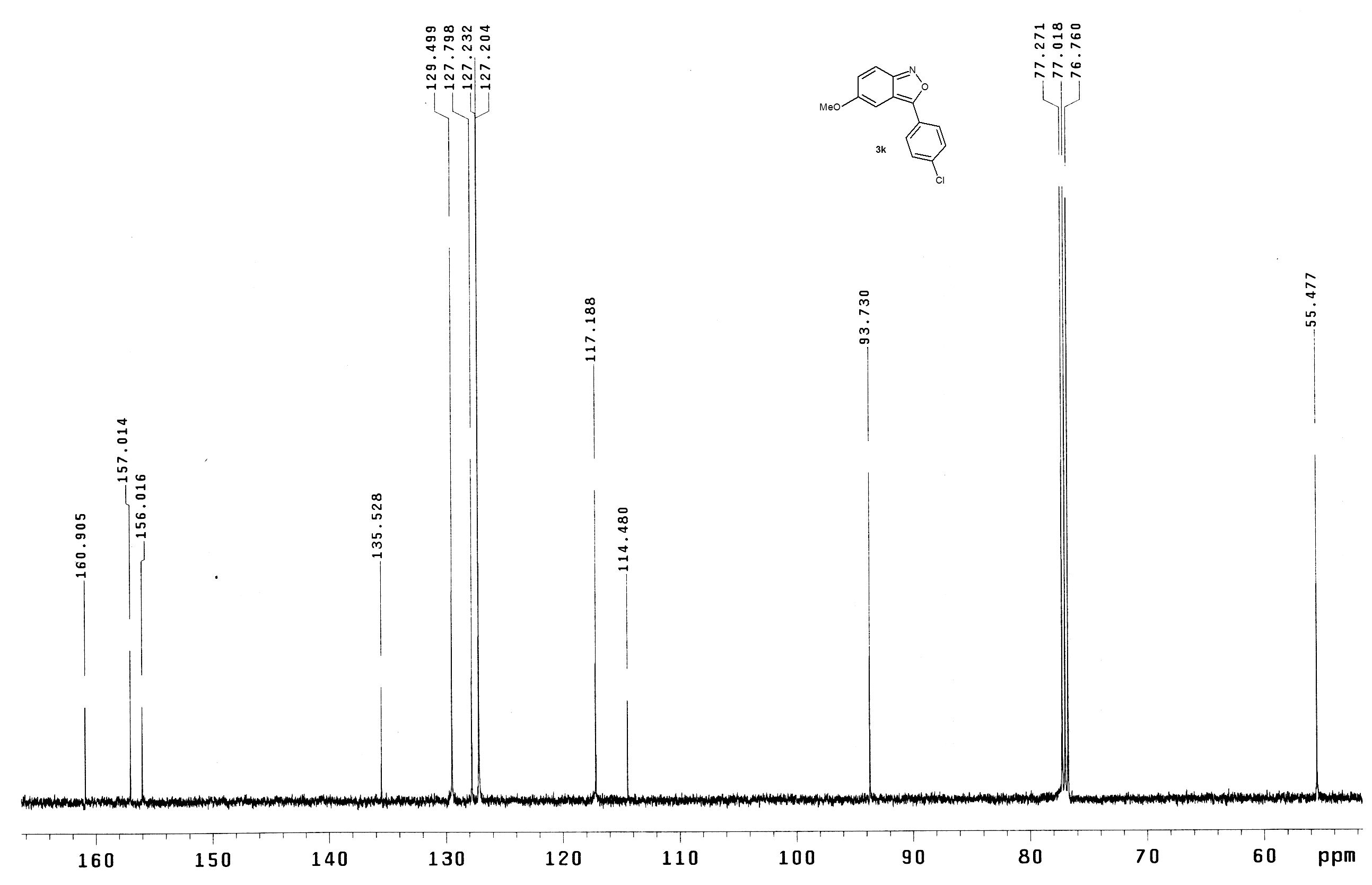


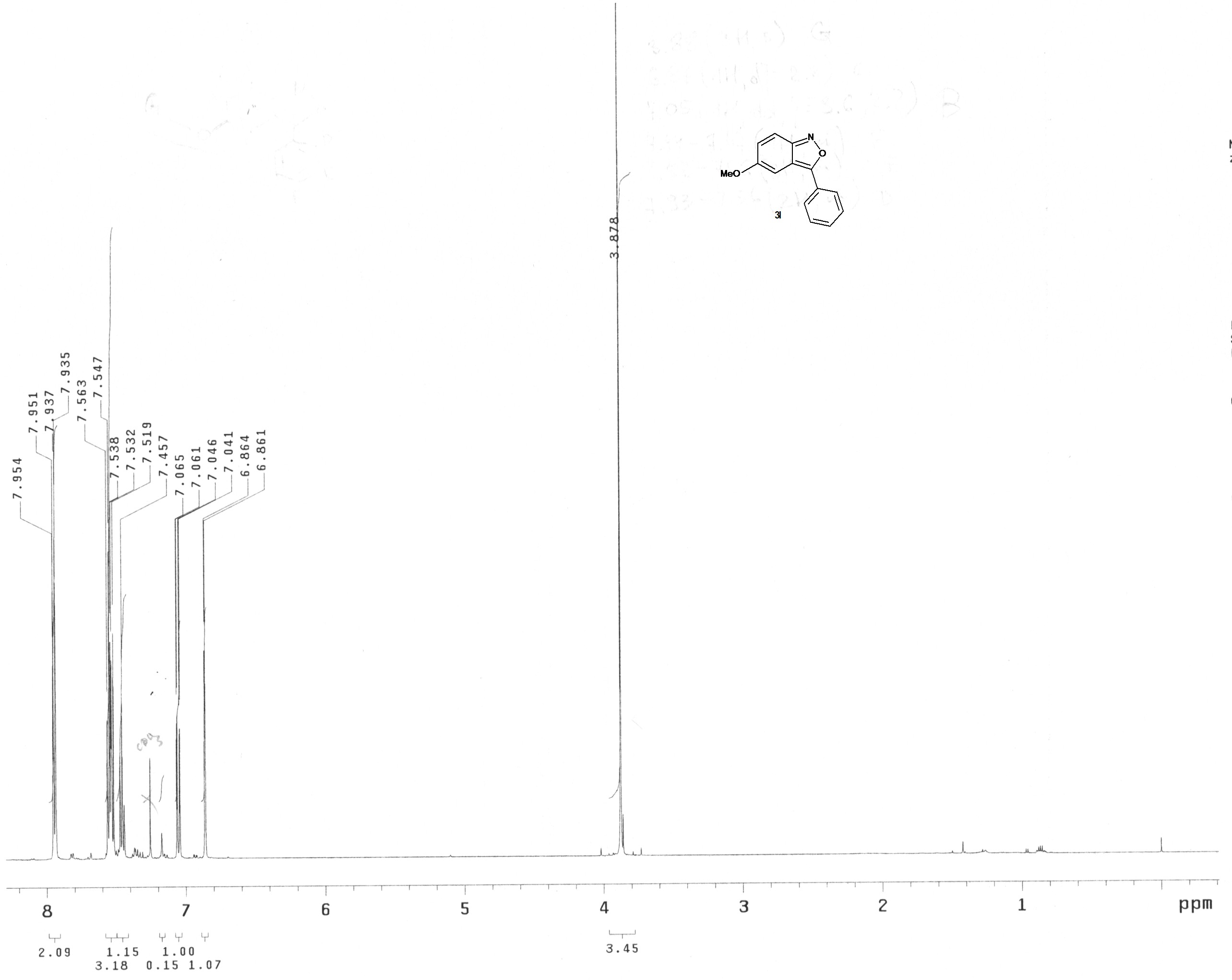

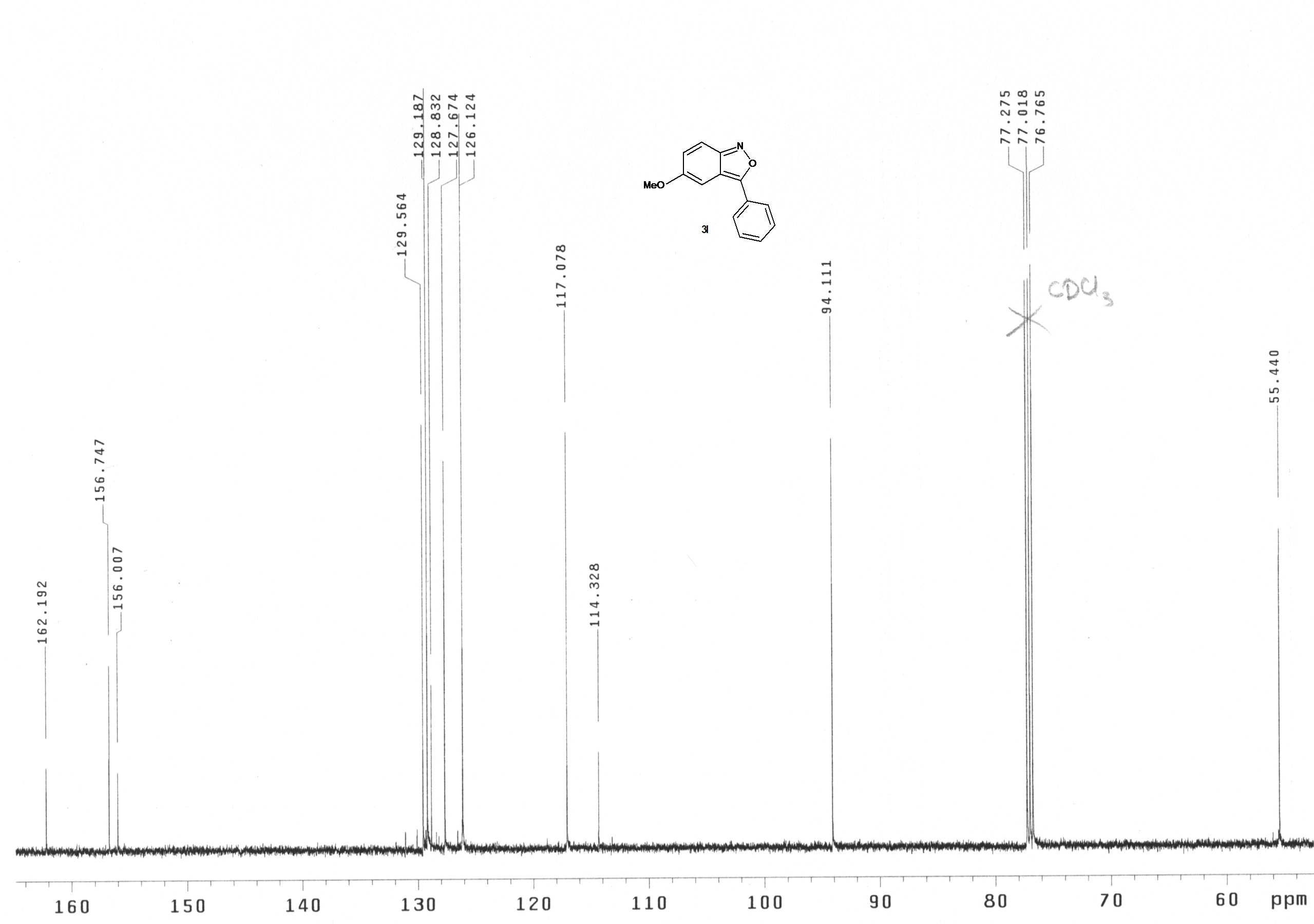


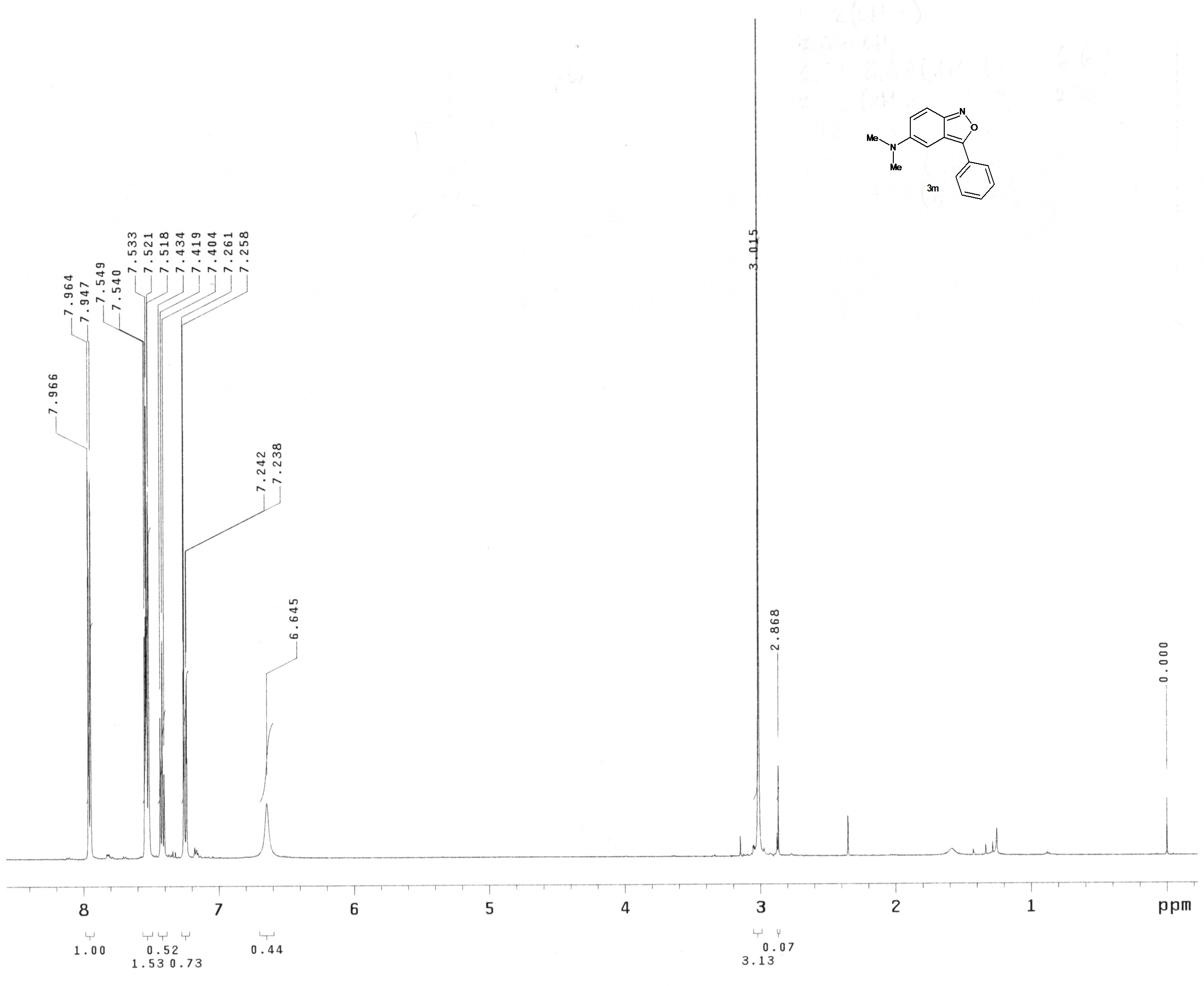


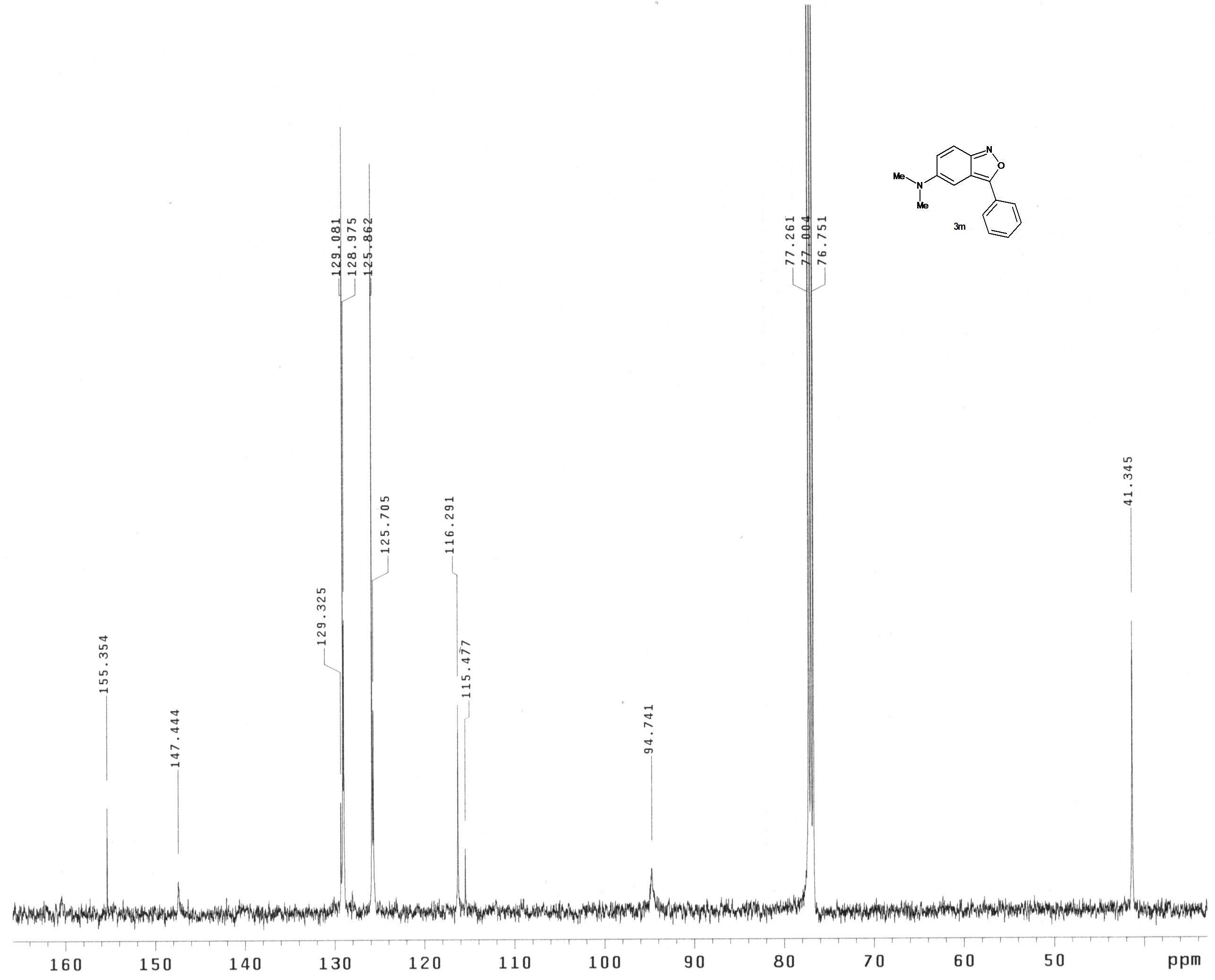

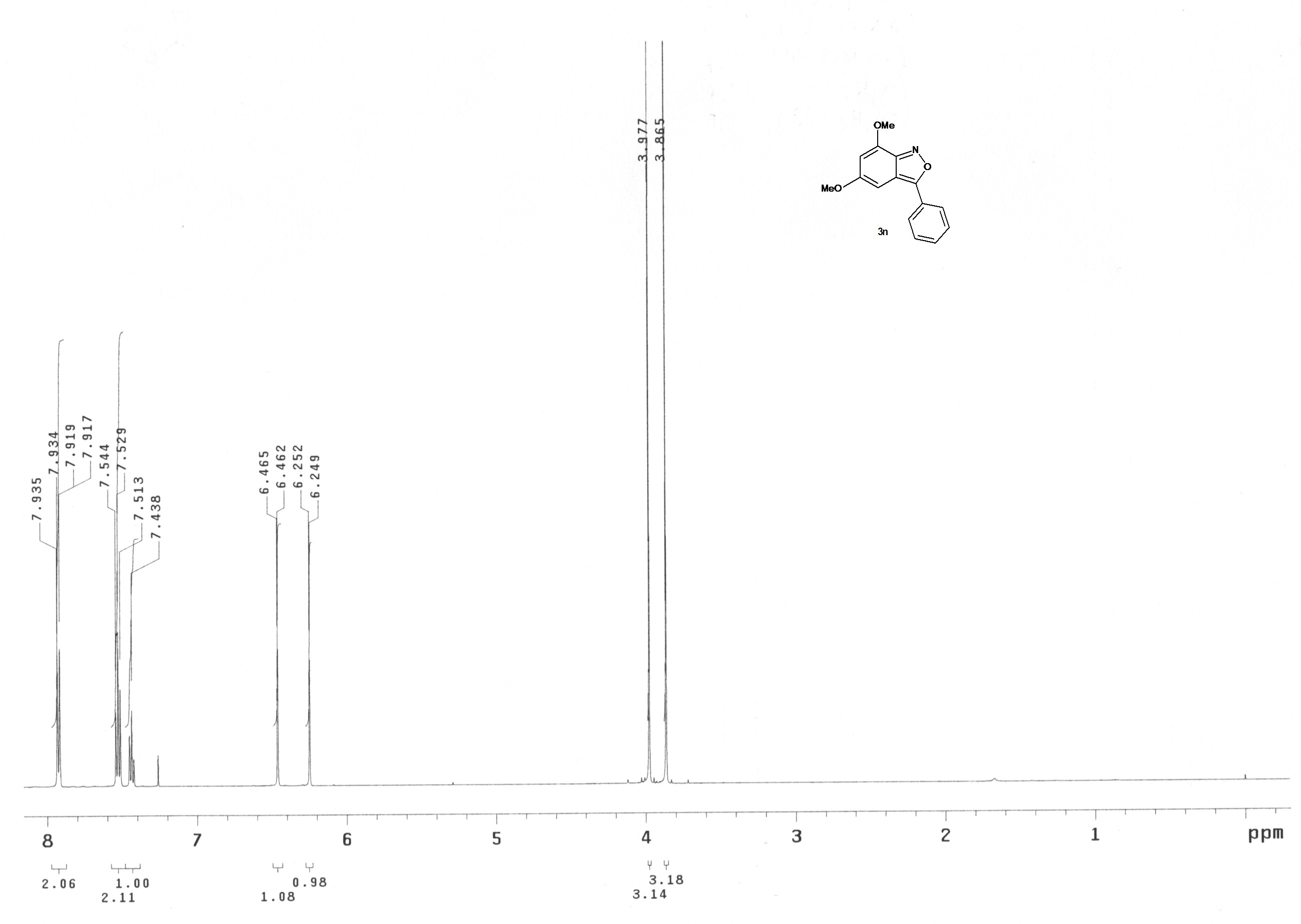

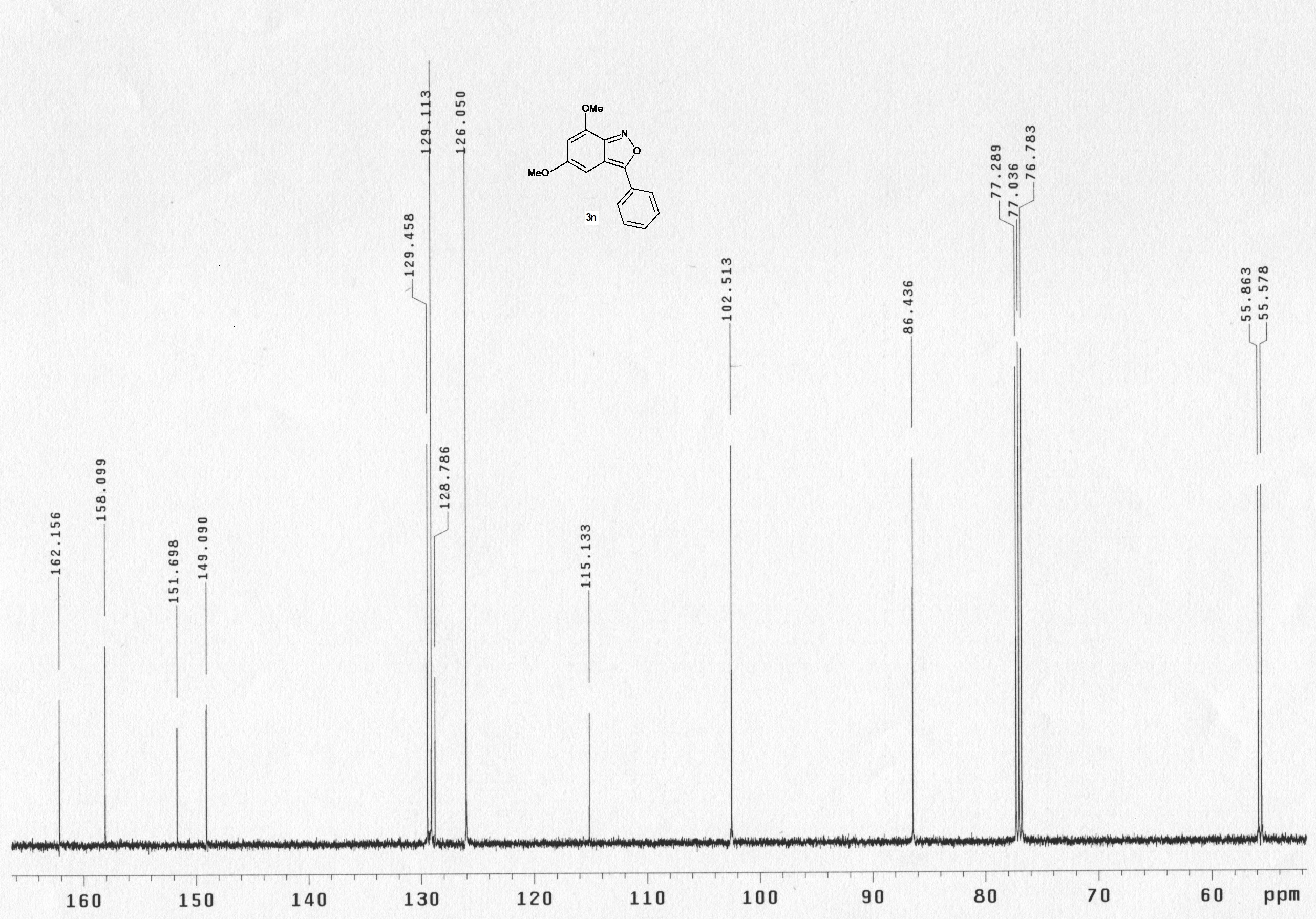

Supplement: Supplementary file 1 — Supplementary Material 1H and 13C spectra of compounds 3a-3n. (doc 6.23mb) [file 11030_2015_9627_MOESM1_ESM.doc]
